# Development, validation, and visualization of a novel nomogram for predicting clinical outcomes of radiotherapy combined with chemotherapy in locally advanced cervical cancer

**DOI:** 10.3389/fonc.2025.1668971

**Published:** 2025-10-08

**Authors:** Nan Jiang, Xiaoxia Ping, Qian Meng, Yuanqing Liu, Xi Wang, Chunhong Hu

**Affiliations:** ^1^ Department of Radiology, The First Affiliated Hospital of Soochow University, Suzhou, Jiangsu, China; ^2^ Department of Radiation Oncology, The First Affiliated Hospital of Soochow University, Suzhou, Jiangsu, China

**Keywords:** chemoradiotherapy, locally advanced cervical cancer, clinical outcomes, machine learning, DWI

## Abstract

**Background:**

Patients with locally advanced cervical cancer (LACC) have been advised to undergo radical chemoradiotherapy. To determine whether local recurrence or distant metastasis (LRDM) will occur in patients with locally advanced cervical cancer (LACC) after chemoradiotherapy, this study aims to develop and validate a model using clinical and radiomic parameters.

**Methods:**

A total of 118 patients with LACC who were treated with radiotherapy combined with chemotherapy were included. They were divided into a training set (n=83) and a validation set (n=35) at an 7:3 ratio. All patients' diffusion-weighted imaging (DWI) images were uploaded to the ITK-SNAP software. Regions of interest (ROIs) were manually delineated, and a radiomic model was constructed using radiomic features by the LightGBM algorithm. A comprehensive model was constructed by integrating clinical and radiomic features and was visualized as a nomogram. The area under the curve (AUC) values were used to evaluate their predictive performance, and Decision curve analysis (DCA) was employed to assess the clinical utility of the predictive models. The calibration curves were used to assess the agreement between predicted and observed outcomes for the LRDM in both cohorts.

**Results:**

Seven variables were finally chosen for modeling using the least absolute shrinkage and selection operator (LASSO) regression analysis. The AUC values for the training and test sets of the DWI radiomic model were 0.789 and 0.785, respectively. AUC values for the training and test sets were 0.897 and 0.889, respectively, for the combined model LGBM-nomogram that used DWI and clinical characteristics. The nomogram worked remarkably well in both the training and test cohorts, as shown by the calibration curves.

**Conclusion:**

The model integrating DWI and clinical features has shown high value in non-invasive prediction of LRDM, which may aid in treatment and prognostication.

## 1 Introduction

Cervical cancer ranks fourth among women globally, making it a serious global health concern (1). Cervical cancer is typically treated with a combination of treatment modalities. For locally advanced disease, including stages IB3 to IVA, synchronous chemoradiotherapy is the standard treatment and works well for many patients. For some patients, the prognosis is poor even with the recommended treatment (2). Despite the advantages of concurrent cisplatin and radiation therapy, 40% of patients have a poor prognosis and recurrence with distant metastases (3). The prognosis and survival duration of patients can be enhanced by early detection of potential distant metastases or local recurrence and modification of appropriate treatment measures.

Existing tumor biomarkers often lack high sensitivity and accuracy in early detection of metastasis progression or recurrence (4–6), recurrence or metastases are often detected during follow-up several months after onset. Traditional MRI is a common imaging method for clinical evaluation of cervical cancer, and DWI is now recommended for initial staging, monitoring of response and evaluation of recurrence (7). Nevertheless, the radiologist's eyes have found only a limited amount of data. The subjective assessment dependent on conventional imaging modalities poses inherent and substantial constraints in the prediction of cervical cancer. The use of tumor morphology based criteria may be limited by the false negative rate (8). Moreover, the interpretation exhibits significant subjectivity and is prone to being influenced by observer individual differences, which limits its accuracy and reproducibility in predicting recurrence and metastasis in LACC. If reliable biomarkers and models were available to early identify patients at high risk of recurrence or metastasis, clinicians could promptly adjust treatment plans (such as dose escalation or additional adjuvant therapy) to address these cases.

Radiomics is a method that numerous quantitative features are extracted and analyzed from radiological images using computer algorithms. In addition to capturing imaging characteristics that are difficult or impossible for the human eye to distinguish, these radiomic features can be translated into mineable data for objective and repeatable evaluation (9). Also, radiomics surpasses traditional imaging tests in terms of early tumor diagnosis and response to treatment, and limitations of other applications, including survival prediction (10) Radiomics can better reflect the heterogeneity of tumors and effectively make up for the shortcomings of traditional methods. In addition, radiomics models can be combined with clinical and molecular data to predict far better than traditional imaging or biomarkers alone. Currently, there is some research on the prediction of metastasis or recurrence for cervical cancer patients showing the importance of developed models (11–14). However, we have not encountered any reports on the use of models based solely on DWI sequences to forecast local recurrence or distant metastasis in patients with locally advanced cervical cancer (LACC). The purpose of this research is to determine whether DWI radiomic features can independently or in combination with clinical features improve the predictive efficacy for recurrence or metastasis in LACC. Furthermore, it seeks to develop a novel DWI-based predictive model for assessing the risk of local recurrence or distant metastasis in LACC patients treated with a combination of radiotherapy and chemotherapy. The combination model LGBM-nomogram created as a result of this study may be able to forecast unfavorable clinical outcomes for LACC, which would improve treatment strategies and increase patient survival rates.

## 2 Materials and methods

### 2.1 Patients

The institutional review board granted approval for this retrospective study (ethical code: 2025606), and written informed consent was not required. Data were collected from patients with stage IB3 to IVA cervical cancer, classified according to the 2018 International Federation of Gynecology and Obstetrics (FIGO) criteria, who were treated at the First Affiliated Hospital of Soochow University between October 2013 and December 2018. Due to their inability to undergo surgery, these patients were treated with a combination of chemotherapy and radiation therapy. Conventional MRI scans were conducted within two weeks before the radical chemoradiotherapy. At our institution, a follow-up examination was performed using CT, MRI, PET, ultrasound, and biopsy three and six months following the conclusion of CRT.

**Figure 1:**
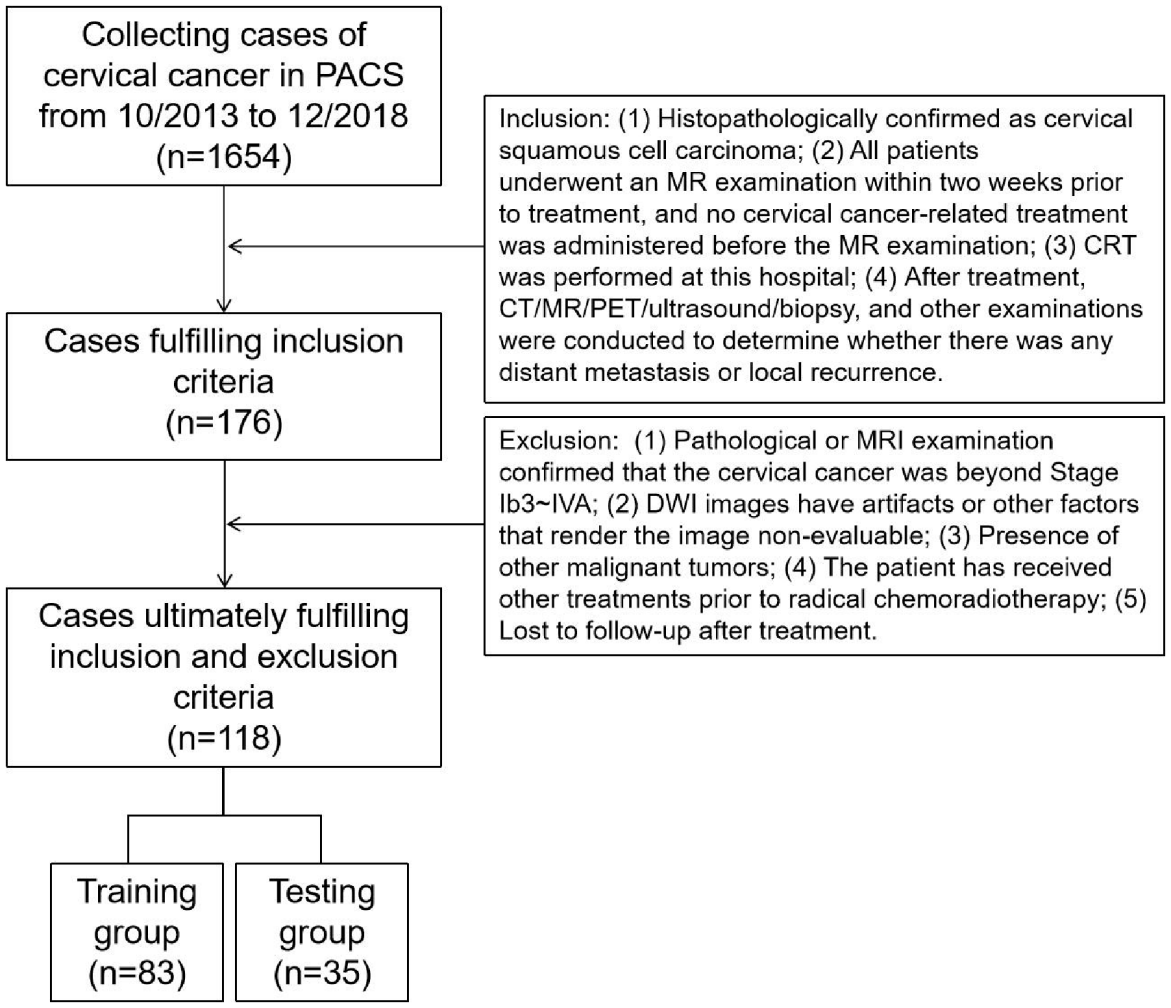
Flowchart for patient screening.

A total of 176 patients met the inclusion criteria, and 118 were ultimately enrolled in the study after applying the exclusion criteria (a flowchart is shown in **Figure 1**). Random assignment was used to assign the patients (age range 32-82 years, median age 52 years, mean age 53.92 ± 11.17 years) to either the training group (83 cases) or the validation group (35 cases) at a 7:3 ratio. Based on the outcomes of tests performed six months following CRT, patients in both the training and test groups were then divided into two groups: those with metastases or recurrences and those without either. Every patient received either standard chemoradiotherapy or radiotherapy plus chemotherapy. The Declaration of Helsinki's tenets were followed throughout the study. Every procedure was carried out in compliance with the regulatory requirements and established institutional protocols. Medical records belonging to patients were anonymized before the review in order to eliminate any identifying information.

### 2.2 MRI protocol

All examinations were performed on 3.0 T MRI scanners, including those manufactured by GE, Philips, and Siemens. The scan range extended from the superior edge of the iliac crest to the inferior edge of the symphysis. The detailed MRI scanning protocols are presented in the **Supplementary Material** and **Supplementary Table S1**.

### 2.3 Clinical features and image analysis

**Figure 2:**
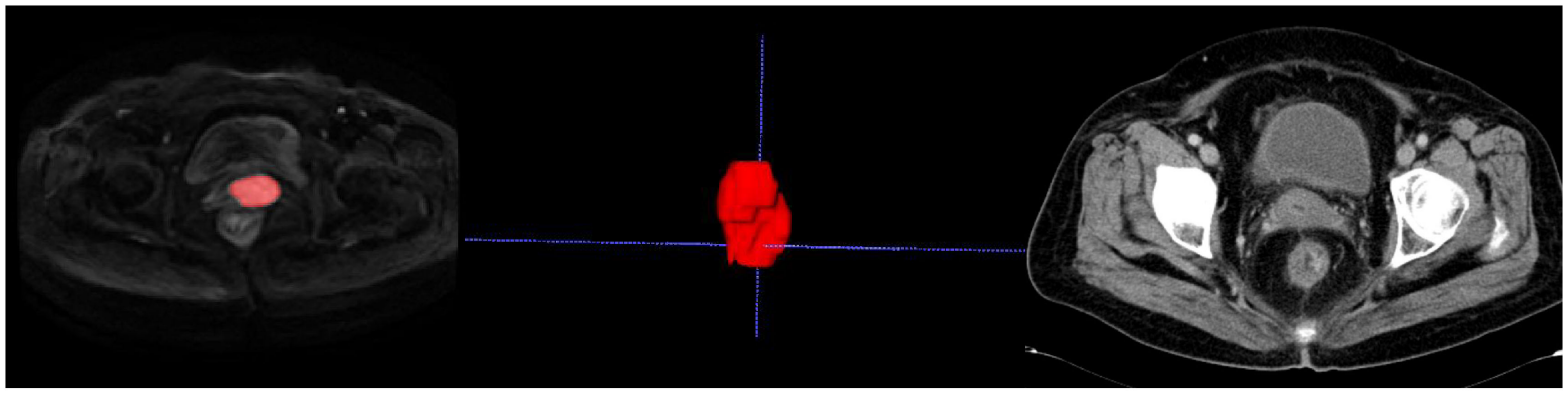
Delineating lesion boundaries of a cervical mass on DWI images in the training group. In the middle is her VOI schematic. This 70-year-old cervical cancer patient showed no recurrence or metastasis in the follow-up review.

**Figure 3:**
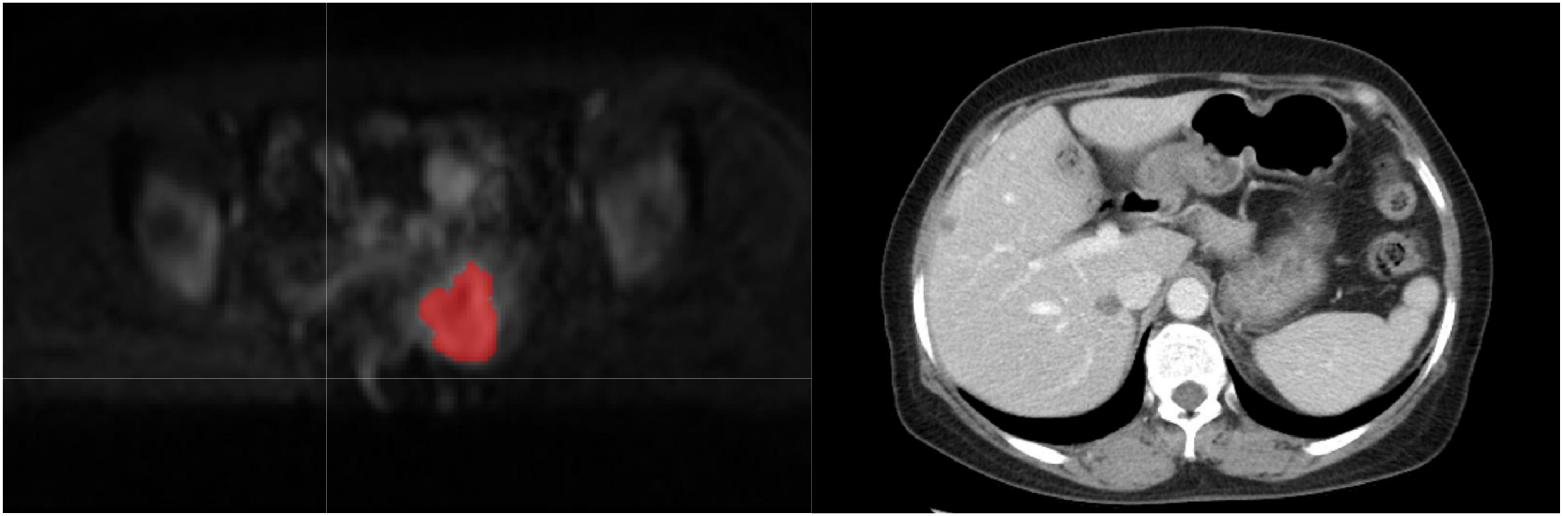
The red portion in the DWI image represents the outlined region of interest (ROI). This 50-year-old cervical cancer patient showed liver metastasis in the follow-up enhanced CT examination.

The medical record system was used to gather clinical information such as age, hypertension, systolic and diastolic blood pressure, labor, abortion times, SCC antigen levels, CA199, CA125, CEA, CA153, CA724, menopausal status, and pregnancy history. The clinical stage was reassessed by a radiologist in collaboration with a radiotherapist, adhering to the 2018 FIGO staging criteria, using original images. The DWI images of cervical cancer were resampled to a voxel size of 1 x 1 x 1 mm³ to standardize voxel spacing. Furthermore, the images were normalized to minimize differences in signal intensity among images captured by various machines. The section with the largest cross-sectional diameter of primary lesions in the axial DWI sequence with b = 1000 s/mm² was utilized to measure tumor diameters by two senior radiologists. The tumor diameters were averaged. In cases of significant discrepancies, the two radiologists resolved the issue together. Prior to image segmentation, the images underwent N4 bias field correction (N4). As shown in **Figures 2** and **3**, axial diffusion-weighted imaging (DWI) images were loaded into the ITK-SNAP program, where regions of interest (ROIs) containing necrosis, bleeding, and degeneration were drawn layer by layer and then combined to create a volume of interest (VOI). PyRadiomics package in Python was used to extract unique radiomic features from the volumes of interest (VOIs) by a senior radiologist. For 30 randomly selected cases, ROI outlining and feature extraction were performed again, and the interobserver agreement was assessed using the intraclass correlation coefficient (ICC). Manual delineation of ROIs is a laborious task subject to inter-observer variability, and semi-automated or automated methods can be used in the future to improve reproducibility. Details regarding the extraction of radiomic features are provided in the **Supplementary Material**.

### 2.4 Treatment regimen and clinical outcome

Chemotherapy could be administered using various combinations. The TP regimen refers to a Taxol and Platinum-based chemotherapy regimen. The TN regimen combines Taxol with Nedaplatin. The TC regimen involves Taxol and Carboplatin. There is also a regimen that involves transitioning from the sole use of Paclitaxel liposome (Lipusu) to a combination platinum chemotherapy. The radiotherapy regimen includes external pelvic radiation and brachytherapy. The external radiation dose ranges from 45 to 50 Gy (1.8-2.0 Gy, administered once daily). For lesions in the paracervical area, an additional dose of 4-10 Gy is administered, and for lymph nodes, an additional 6-10 Gy. The completion of external beam radiotherapy is followed by brachytherapy. Point A serves as the reference point, with a total dose of 21-30 Gy, delivered in fractions of 5-7 Gy, once weekly. The clinical outcomes of LACC were independently assessed by a radiologists and a radiation oncologist based on pre-chemoradiotherapy MRI images and six-month follow-up results. If discrepancies arose, they would deliberate and arrive at a consensus. The disappearance of the mass at the three-month follow-up and its reappearance at the six-month follow-up were defined as local recurrence. The newly added metastases include organ metastases and lymph node metastases. Patients with local recurrence or distant metastasis (LRDM) were classified as the positive group, while those with no evidence of LRDM detected on CT/MRI/PET/ultrasound/biopsy 6 months after completing treatment were categorized as the negative group.

### 2.5 Statistical analysis

SPSS 22.0 and Python 3.8.0 software were utilized for statistical analysis, with a probability value less than 0.05 considered significant. The ICC was employed to assess the stability of parameters, indicating high consistency between two measurements when the ICC exceeds 0.7. Dimensionality reduction using LASSO regression was performed on the radiomics features of the training group with the Python scikit-learn package. The selection of clinical predictors was based on the results of univariate and multivariate logistic regression analysis, combined with clinical experience and relevant literature. The researchers used radiomic features and clinical features to construct a combination model for predicting local recurrence or distant metastasis after CRT treatment of cervical cancer. The prediction model was subsequently introduced to the test group to further validate its diagnostic efficacy. The Hosmer-Lemeshow test was used to assess the fit of the prediction model, while the calibration curve was used to evaluate the predictive performance of the nomogram. The Decision curve analysis (DCA) was used to assess the clinical value of the nomogram. And the discriminatory power for LRDM in LACC was calculated by Receiver Operating Characteristic Curve (ROC).

## 3 Results

### 3.1 Clinical characteristics and measurement results of MRI

A total of 118 patients were enrolled in this study, with 59 in the metastasis or recurrence group and 59 in the group with no evidence of local recurrence or metastasis. The clinical features of the training and test groups, including age, hypertension, systolic and diastolic blood pressure, pregnancy history, labor, abortion timing, SCC antigen levels, CA199, CA125, CEA, CA153, CA724, tumor diameter, FIGO stage, and menopausal status, are detailed in **Table 1**. Most results lacked statistical significance, suggesting that the groupings were randomized and reasonable. In terms of radiomics parameters, both radiologists achieved good interobserver agreement, with interclass correlation coefficients (ICCs) ranging from 0.74 to 0.98.

**Table 1:** Clinical factors and MRI measurement results of patients in training and test groups.

Characteristics	Train-all	Recurrence or metastasis	No evidence	*P*-value	Test-all	Recurrence or metastasis	No evidence	*P*-value
Age	53.76±11.29	54.68±12.63	52.83±9.82	0.658	54.54±10.89	53.17±10.36	55.92±11.69	0.548
Systolic pressure	123.61±15.95	119.94±13.66	127.28±17.33	0.025	123.79±16.43	118.08±12.50	129.50±18.35	0.132
Diastolic pressure	78.13±11.19	76.11±8.93	80.15±12.85	0.161	78.58±14.76	74.83±8.45	82.33±18.81	0.451
Pregnancy	2.44±1.50	2.40±1.65	2.47±1.35	0.523	2.29±1.04	2.42±1.24	2.17±0.83	0.466
Labor	1.66±1.09	1.70±1.27	1.62±0.90	0.781	1.46±0.83	1.25±0.87	1.67±0.78	0.133
Abortion	0.78±1.04	0.70±1.02	0.85±1.06	0.357	0.83±0.92	1.17±1.03	0.50±0.67	0.101
SCCa(ng/ml)	9.00±12.67	10.58±14.50	7.42±10.47	0.108	10.12±18.48	12.53±13.51	7.72±22.78	0.004
CA199(U/ml)	18.29±23.99	18.25±23.63	18.34±24.61	0.777	10.07±12.46	5.34±4.01	14.79±16.11	0.069
CA125(U/ml)	34.70±79.18	33.49±55.82	35.91±97.75	0.304	40.25±67.53	33.56±25.32	46.94±93.79	0.285
CEA(ng/ml)	7.39±11.45	10.75±14.97	4.03±4.23	0.034	10.17±19.39	11.92±17.29	8.43±21.93	0.341
CA153(U/ml)	14.41±13.02	16.85±17.15	11.97±6.01	0.072	12.76±5.44	13.29±6.09	12.23±4.92	0.645
CA724(U/ml)	5.49±6.76	4.87±4.72	6.11±8.32	0.871	6.29±9.81	8.19±13.39	4.39±3.76	0.665
Tumor diameter(mm)	44.31±17.73	45.64±18.71	42.98±16.78	0.469	43.19±18.99	53.17±17.49	33.21±15.21	0.007
Hypertension				0.100				0.109
0	56(67.47)	31(75.61)	25(59.52)		25(71.43)	15(83.33)	10(58.82)	
1	27(32.53)	10(24.39)	17(40.48)		10(28.57)	3(16.67)	7(41.18)	
Menopause				0.531				0.773
0	27(32.53)	12(29.27)	15(35.71)		9(25.71)	5(27.78)	4(23.53)	
1	56(67.47)	29(70.73)	27(64.29)		26(74.29)	13(72.22)	13(76.47)	
Stage				0.682				0.001
1	17(20.48)	10(24.39)	7(16.67)		7(20.00)	0	7(41.18)	
2	40(48.19)	19(46.34)	21(50.00)		16(45.71)	10(55.56)	6(35.29)	
3	15(18.07)	8(19.51)	7(16.67)		3(8.57)	0	3(17.65)	
4	11(13.25)	4(9.76)	7(16.67)		9(25.71)	8(44.44)	1(5.88)	

**Table 1** displays the clinical characteristics and MRI measurement results. The outcomes of the univariate logistic regression analysis are detailed in **Table 2**. Notably, only CEA demonstrated significant differentiation capabilities for local recurrence and distant metastases subtypes.

**Table 2:** Univariate logistic regression analysis of clinical and MRI results to differentiate local recurrence and distant metastases in the training cohort.

Characteristics	Log(OR)	Lower 95%CI	Upper 95%CI	OR	OR lower 95%CI	OR upper 95%CI	*p*_value
Age	-0.001	-0.007	0.006	0.999	0.993	1.006	0.87
Hypertension	0.46	-0.147	1.066	1.583	0.863	2.904	0.213
Systolic pressure	0	-0.002	0.003	1.001	0.998	1.003	0.775
Diastolic pressure	0.001	-0.004	0.005	1.001	0.996	1.005	0.804
Menopause	-0.095	-0.51	0.32	0.909	0.6	1.377	0.706
Pregnancy	0.008	-0.111	0.127	1.008	0.895	1.135	0.914
Labor	-0.022	-0.193	0.15	0.979	0.824	1.162	0.835
Abortion	0.09	-0.175	0.355	1.094	0.839	1.426	0.578
SCCa	-0.014	-0.036	0.009	0.987	0.965	1.009	0.33
CA199	0	-0.011	0.011	1	0.989	1.011	0.988
CA125	0	-0.004	0.004	1	0.996	1.004	0.892
CEA	-0.048	-0.085	-0.011	0.953	0.919	0.989	0.032
CA153	-0.014	-0.033	0.005	0.986	0.968	1.005	0.235
CA724	0.017	-0.023	0.057	1.017	0.977	1.059	0.494
Stage	0.018	-0.123	0.159	1.019	0.884	1.172	0.83
Tumor diameter	-0.001	-0.008	0.006	0.999	0.992	1.006	0.787

### 3.2 Radiomics features extraction and selection

**Figure 4:**
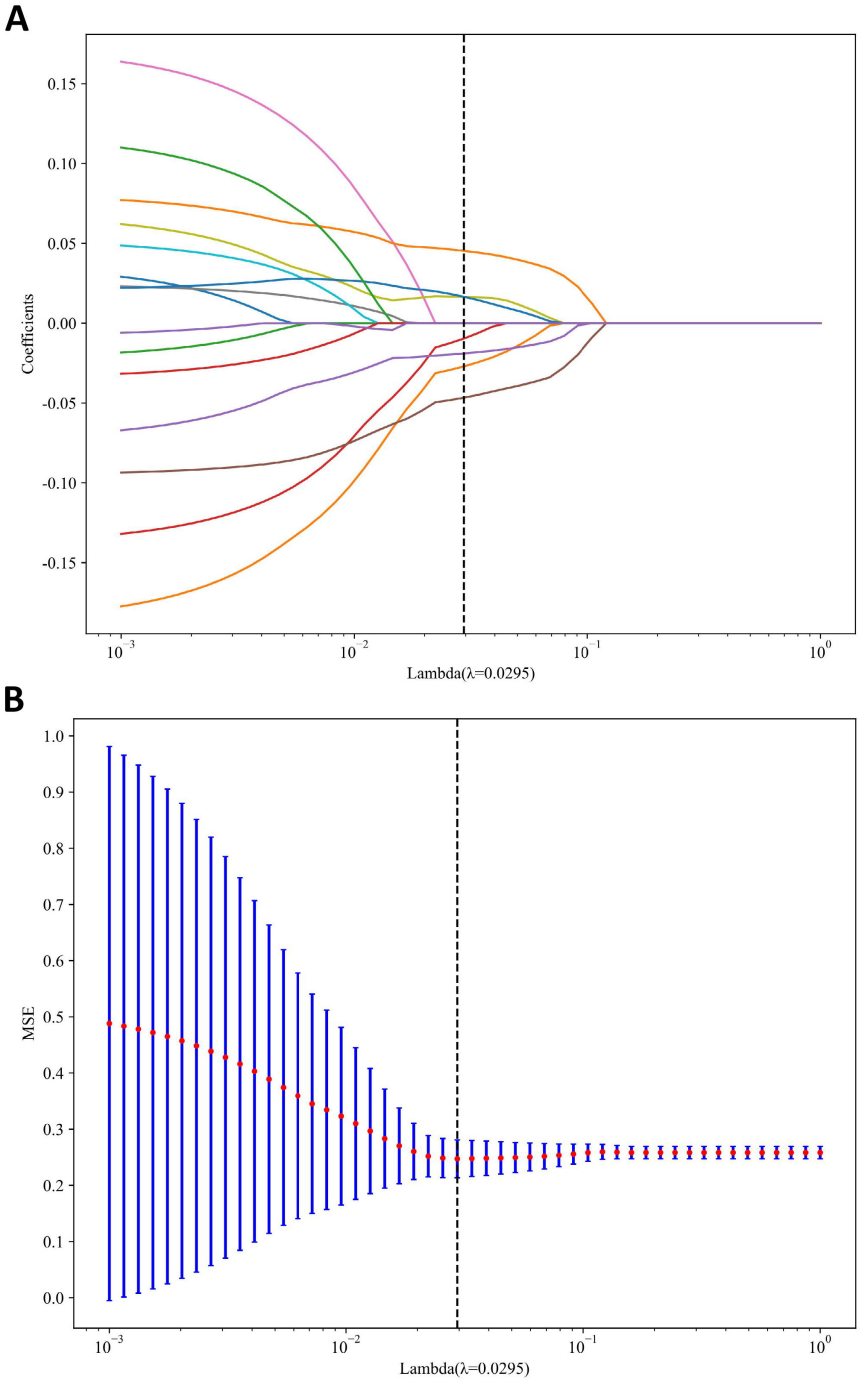
MRI radiomics feature selection utilizing the Least Absolute Shrinkage and Selection Operator (LASSO) algorithm. **(A)** LASSO coefficient profiles of diffusion-weighted imaging (DWI) radiomics features. **(B)** Mean square error path derived from tenfold cross-validation for the DWI radiomics feature selection process.

**Figure 5:**
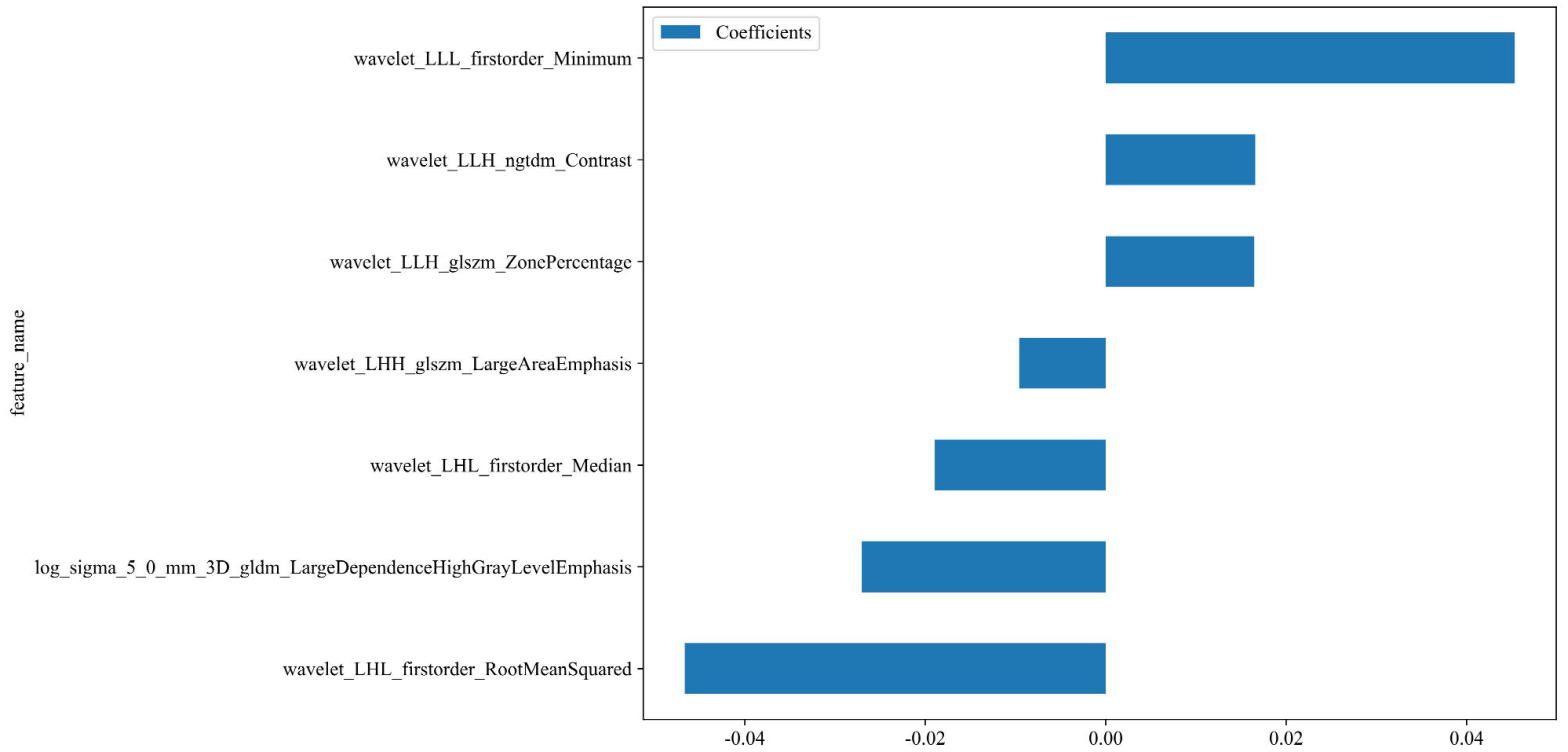
The weight of the features that are ultimately selected. Rad-score=0.5112781954887218 -0.027037 * log_sigma_5_0_mm_3D_gldm_LargeDependenceHighGrayLevelEmphasis -0.009586 * wavelet_LHH_glszm_LargeAreaEmphasis -0.018953 * wavelet_LHL_firstorder_Median -0.046651 * wavelet_LHL_firstorder_RootMeanSquared +0.016458 * wavelet_LLH_glszm_ZonePercentage +0.016571 * wavelet_LLH_ngtdm_Contrast +0.045326 * wavelet_LLL_firstorder_Minimum.

A total of 1198 radiomic features were extracted from the ROIs of DWI images. These features include geometric shape features, texture features and intensity features. Texture features were extracted using various methods, including the Gray-Level Co-occurrence Matrix (GLCM), the Gray-Level Run Length Matrix (GLRLM), the Gray-Level Size Zone Matrix (GLSZM), and other methods. The distribution of radiomics features across different classifications was presented in **Supplementary Figure S1**. First, we normalize the data by subtracting the average value and dividing by the standard deviation using the Z-score method. Z-score normalization effectively eliminated scale differences among features, ensuring that each feature contributed to subsequent modeling with comparable weight when using machine learning algorithms, thereby improving model convergence and stability. However, it is important to acknowledge that Z-score normalization primarily addresses the issue of feature scale variation within a single dataset. Its ability to correct for systematic distributional differences introduced by different scanners or acquisition protocols is limited. It cannot correct for systematic shifts in mean and variance between different batches of data. Then, we conducted the Mann-Whitney U test and feature screening for all radiomic features. Only those with a p-value less than 0.05 were retained. In the selection process, Pearson correlation analysis was employed to retain one of the two features with a correlation coefficient greater than or equal to 0.7. In order to retain the ability to depict features to the greatest extent, we use greedy recursive deletion strategy for feature filtering, that is, the feature with the greatest redundancy in the current set is deleted each time. After this, fifteen features were subjected to LASSO regression analysis, which ultimately screened out seven for modeling. To determine the optimal λ, we employed 10-fold cross-validation using a minimum criteria approach, where the final λ value resulted in the minimum cross-validation error. **Figure 4** illustrates that the optimal lambda value is 0.0295. The features with nonzero coefficients that were retained were utilized for fitting the regression model and were combined to form a radiomics signature. We calculated a radiomics score for each patient through a linear combination of the retained features, weighted by their respective model coefficients. **Figure 5** shows the weight of each feature.

### 3.3 Radiomics model development and evaluation

**Figure 6:**
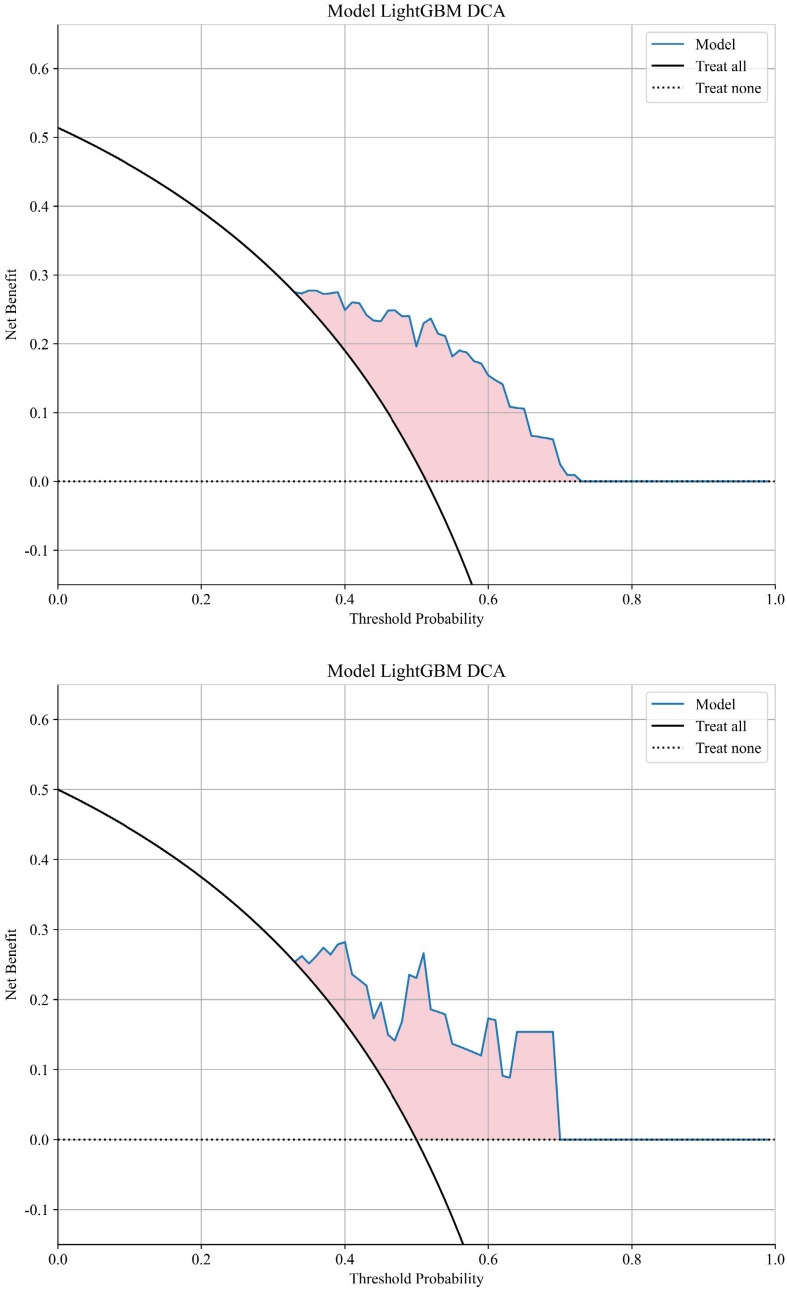
DCA curve in the training cohort and in the testing cohort. DCA, decision curve analysis.

The AUC for distinguishing between the recurrence or metastasis group and the other group using a machine learning algorithm is 0.789 in the training cohort and 0.785 in the validation cohort. The LightGBM model exhibited superior performance in terms of AUC and was consequently employed to differentiate whether there will be distant metastasis or local recurrence after chemoradiotherapy. Visualization of sample predictions by LightGBM algorithm in training set and validation set was shown in **Supplementary Figure S2**. The DCA graph shows the net benefit (Net Benefit) of the LightGBM model at different threshold probabilities to evaluate the clinical utility of the model. The model curve (Blue solid line) of the training set and validation set (**Figure 6**) is higher than that of "Treat all" (Black solid line) and "Treat none" (Black dashed line) in most threshold ranges, indicating that the model has clinical utility.

### 3.4 Clinical model

The construction of a clinical signature is nearly identical to that of a radiomic signature. Initially, features for constructing the clinical signature, CEA, were chosen based on baseline statistics with a p-value of less than 0.05. We also added age, tumor diameter, Figo stage, CA125, and SCCa to the clinical model based on relevant literature and clinical experience. The same machine learning model was used in the construction of the clinical signature.

### 3.5 Development and assessment of the nomogram

**Figure 7:**
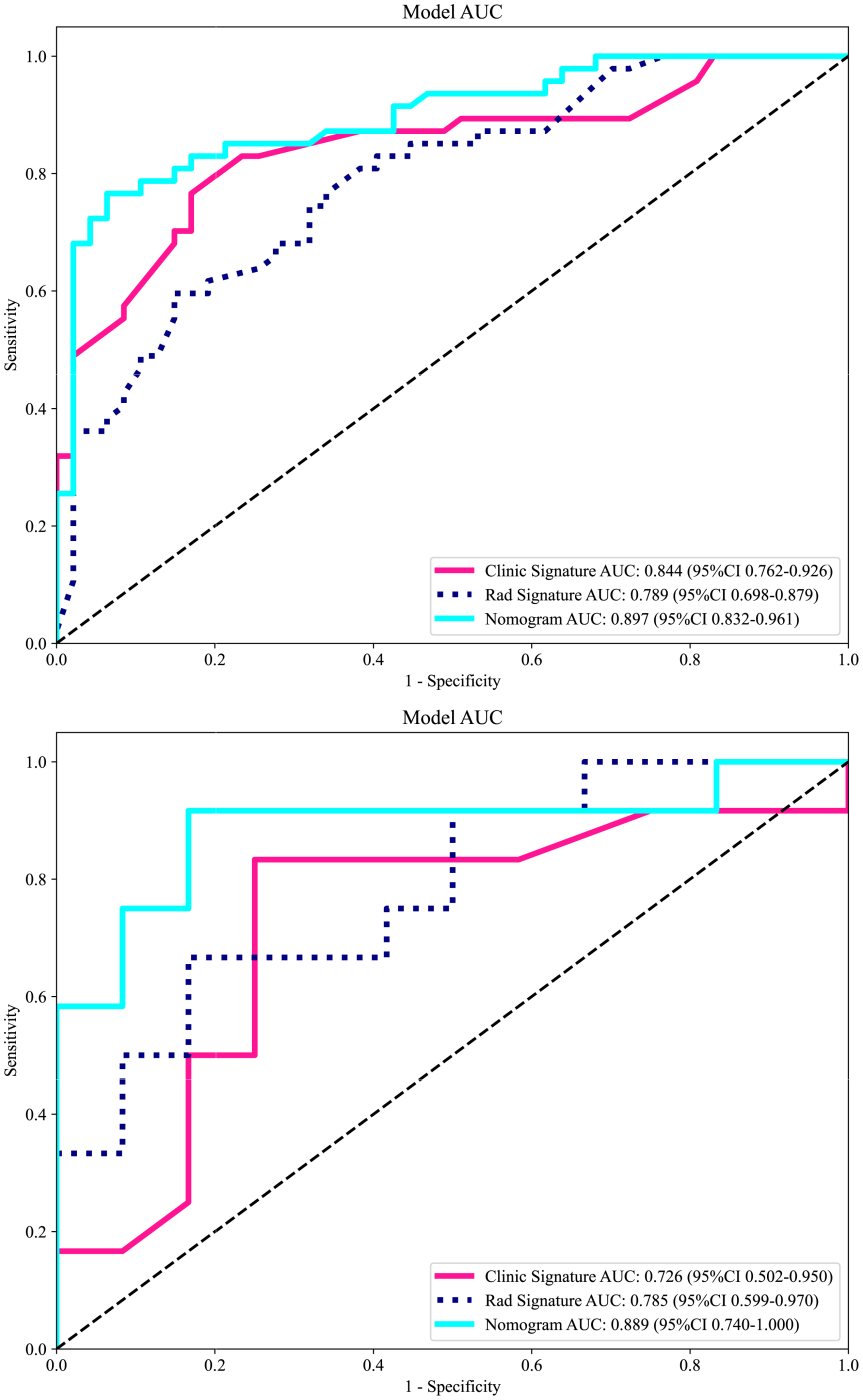
The receiver operating characteristic (ROC) curves of the DWI radiomics model, clinical model, and combined model in the training cohort and in the validation cohort. Regarding the prediction of LRDM, the combined model exhibited the best performance in both the training and test sets. LRDM, local recurrence or distant metastasis.

**Figure 8:**
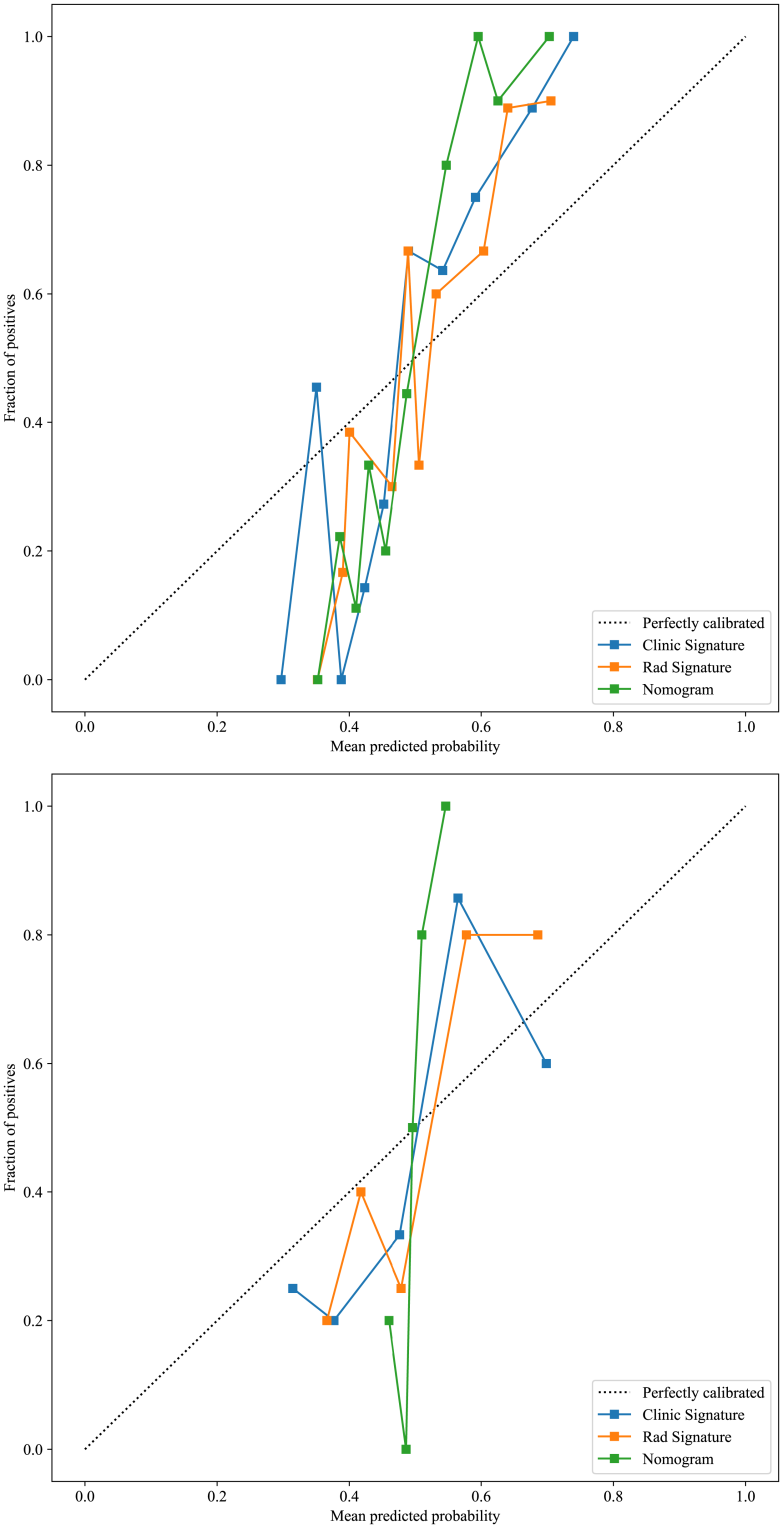
The calibration curves for the training and test cohorts, along with the Hosmer-Lemeshow test results. The calilbration curves of this model were obtained by resampling 1000 times. The LGBM-nomogram calibration curves indicate a good agreement between predicted and observed outcomes for LRDM in both cohorts. These results demonstrate that the nomogram performs exceptionally well in both the training and test cohorts.

**Figure 9:**
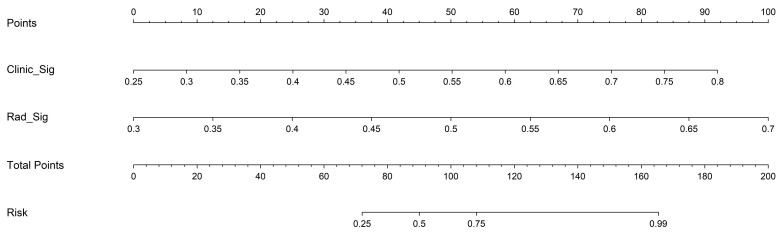
The LGBM-nomogram for clinical use.

Based on radiomics and clinical characteristics, we developed a combined model named LGBM-nomogram. This model offers a quantitative representation of each variable and enables individualized calculations of the patient's total risk score, which correlates with the likelihood of metastasis or recurrence. ROC curves were utilized to evaluate the discriminative ability of the models. In the training group, the AUC value for the radiomics model was 0.789, and for the clinical model, it was 0.844. The AUC for the nomogram model, which incorporates both radiomics and clinical features, was 0.897 (refer to **Figure 5**). In the test group, the AUC for the radiomics model was 0.785, and for the clinical model, it was 0.726. The AUC for the nomogram model, which combines radiomics and clinical features, was 0.889 (refer to **Figure 7**). The model's calibration can be verified using the calibration plot, which demonstrates excellent agreement between the predicted and actual likelihood of recurrence or metastasis (**Figure 8**). DCA was used to evaluate the clinical utility of the predictive models. The results indicated that the net benefit is substantial. The fusion model was depicted in the nomogram (**Figure 9**).

## 4 Discussion

The early signs of cervical cancer are frequently vague and atypical. By the time cancer is diagnosed, it has usually progressed to a locally advanced stage. Chemoradiotherapy is used to treat locally advanced cervical cancer. One-third of patients who undergo chemoradiation followed by brachytherapy for locally advanced disease will experience a recurrence (15). Targeted therapies can enhance the uptake of chemotherapy drugs. Nonetheless, there are still cases with limited benefits, representing an unmet need. Therefore, early assessment of the undesirable clinical outcome of patients is essential for the selection of individualized treatment plans, which is crucial for improving survival rates.

The current assessment of cervical cancer relies on morphological imaging modalities, including CT, MRI, and ultrasound. These conventional techniques pose challenges for direct visual assessment in predicting local recurrence or distant metastasis prior to treatment. Radiomics, unlike traditional imaging, can enable the high-throughput extraction, quantification, and analysis of subvisual features from medical images, thereby capturing intratumoral heterogeneity and reflecting underlying genetic variations. The heterogeneity detected by radiomics in LACC may be associated with different clinical outcomes. Predictive models based on radiomic features show potential for identifying patients at increased risk of local recurrence or distant metastasis following chemoradiation. Consequently, for these patients, therapeutic strategies could be optimized and follow-up protocols intensified, thereby potentially improving overall clinical management and prognosis.

Machine learning and deep learning methods can assist in the detection of cervical cancer, definitive diagnosis, staging, prognosis prediction, and the assessment of therapeutic sensitivity, among other applications (16–19). Previous studies have confirmed the feasibility of predicting sensitivity or response to concurrent chemoradiotherapy when combined with clinical prognostic factors (20–22). There have also been studies investigating the prediction of clinical outcomes for cervical cancer, using either multiple MRI sequences or focusing on a single outcome. However, no study has so far dealt with the prediction and discussion of local recurrence or distant metastasis by using a single sequence combined with clinical data. We have successfully developed the LGBM model, which is capable of predicting local recurrence or distant metastasis in locally advanced cervical cancer. This model is an efficient machine learning algorithm, based on the gradient boosting framework, and is used for ranking, classification, and other machine learning tasks. It is less prone to generalization issues than earlier machine learning models and yields significantly greater accuracy, surpassing that of any other boosting algorithms (23). It is robust against noise and outliers and does not easily overfit when faced with complex data distributions. The LGBM employs a histogram-based decision tree algorithm, which can effectively process large-scale data while maintaining high accuracy. The AUC value in the LGBM model reached 0.897, indicating a high level of accuracy and prediction efficiency. This model, which utilized easily obtainable clinical data, laboratory measurements, and DWI sequences, can serve as a valuable decision-support tool to inform and guide treatment planning in clinical practice. It can assist in identifying high-risk groups for local recurrence or distant metastases of LACC, enabling timely adjustments to clinical decision-making and proactive interventions. Our results indicate that the clinical model, developed using the LightGBM machine learning algorithm, achieved an AUC of 0.844 on the training set. The radiomics model followed with an AUC of 0.789, and the combined clinical-imaging model reached an AUC of 0.897. In the validation set, the clinical model demonstrated an AUC of 0.726, the radiomics model achieved an AUC of 0.785, and the combined clinical-imaging model attained an AUC of 0.889.

A review study has found that elevated pretreatment levels of squamous cell carcinoma antigen (SCC Ag) are associated with poor survival rates in patients with cervical cancer who undergo definitive concurrent chemoradiotherapy (24). Hou et al. (25) studied that SCC-Ag, CEA, CA125, PLT, FIB and CRP were identified as important predictors of lymph node metastasis in IA2-IIA1 cervical cancer patients. According to clinical experience and relevant literature (26), we know that age is an important factor in the clinical prognosis of cervical cancer (CC) patients. This study failed to identify a significant role of age in influencing local recurrence or distant metastasis in LACC patients following CRT treatment. A possible explanation may lie in the fact that this was a single-center retrospective study, which is susceptible to selection bias. Middle-aged and older patients who sought medical care might have constituted a self-selected group with stronger health-seeking intentions and comparatively better physical condition. According to research (27) by Brodeur et al., the FIGO 2018 classification and MRI tumor diameter were predictors of recurrence in CC. It was found that the p-values for FIGO stage and tumor diameter were greater than 0.05 in this study. We speculate that the possible reasons may include the relatively small sample size, particularly the insufficient number of advanced-stage (Stage III/IV) cases and patients with larger tumors, resulting in inadequate statistical power to detect prognostic differences among different stages and tumor size groups. Additionally, the short follow-up period might have been insufficient to observe the occurrence of adverse outcomes, leading to current data that cannot fully reflect long-term prognostic value. So we added age, tumor diameter, Figo stage, CA125, and SCCa to construct our clinical model. A study found that pre-treatment CEA levels greater than 10 ng/ml can predict a poor prognosis for advanced cervical squamous cell carcinoma (28). In our research, we also found that CEA exhibited significant statistical significance in distinguishing between positive and negative groups through multivariate logistic regression analysis. In both the training and validation groups, 10 ng/ml can indeed be used as an intercept to distinguish between positive and negative set.

This study employed multiple screening methods to identify the final characteristics for building the radiomics model. The features with high coefficients selected by LASSO are utilized for model building: 'log_sigma_5_0_mm_3D_gldm_LargeDependenceHighGrayLevelEmphasis', 'wavelet_LHH_glszm_LargeAreaEmphasis', 'wavelet_LHL_firstorder_Median', 'wavelet_LHL_firstorder_RootMeanSquared', 'wavelet_LLH_glszm_ZonePercentage', 'wavelet_LLH_ngtdm_Contrast', 'waveletLLL_firstorder_Minimum'. Of the seven features we ultimately selected, six were based on the wavelet transform, and one was based on the Gaussian filter. Among them, the feature with the highest weight is the RootMeanSquare (RMS) among the first-order features. RootMeanSquare is also important in predicting 5-year progression-free survival (PFS) in high-risk prostate cancer (29). RMS is calculated as the square root of the mean of all squared intensity values and serves as another measure of the magnitude of image values. This feature is susceptible to volume confounding. The feature 'Minimum,' which has a weight ranking among the primary features, was the second most significant. In a study of sensitivity prediction for concomitant chemoradiotherapy in cervical cancer, the feature Minimum was also used to build the model (20). This indicates that the shortest path, frequently overlooked morphologically, may be related to subsequent clinical outcomes for patients, thus meriting further attention. Ranking third in terms of weight is the LargeDependenceHighGrayLevelEmphasis (LDHGLE). It employs a linear smoothing filter, which effectively preserves the edge information of the image while eliminating noise. This feature is used to measure the joint distribution of large dependence with higher gray-level values. These radiomics features are predictive of tumor prognosis, and First-order characteristics plays an important role in this regard. One study have found that five features, including ngtdm Contrast, FirstorderRootMeanSquared, LargeDependenceHighGrayLevelEmphasis, could be used as survival biomarkers for head and neck squamous cell carcinoma (30). Three of the characteristics are consistent with us, indicating that these characteristics are more important for the prognosis of squamous cell carcinoma. In the study of Cui et al., LargeDependenceHighGrayLevelEmphasis was independently associated with complete pathological response(CPR) (31). This feature is also very critical and has the third highest weight in our model.

Distant metastasis poses a significant burden on patients with locally advanced cervical cancer, with the lungs, distant lymph nodes, and bones being the most common sites (32). Cervical cancer recurs in 25% to 61% of women, depending on the stage at initial presentation (33). In our findings, we identified 59 instances of distant metastasis and local recurrence. Specifically, there were 12 cases of recurrence, 15 instances of lung metastasis, 1 case of pancreatic metastasis, 9 cases of bone metastasis, 3 instances of liver metastasis, and 20 cases of metastasis in the supraclavicular, mediastinal, retroperitoneal, and inguinal lymph nodes, as well as 2 cases of abdominal pelvic metastasis. In this study, treatment is not limited to curative chemoradiotherapy; some patients receive compensatory chemotherapy following radiotherapy. This subset of cases is relatively small, and no subgroup analysis has been conducted. Further exploration will be undertaken as more patients are enrolled in the future.

Our results show that radiomics models based solely on DWI sequences, clinical features, and nomogram can be used to predict local recurrence and distant metastases. In the field of gynecological oncology, the European Society of Urogenital Radiology (ESUR) recommends the use of DWI and dynamic contrast enhanced imaging (DCE-MRI) for local staging of cervical cancer (34). The DLRN model was developed by integrating independent clinical factors using T2WI and DWI images. It can predict and stratify recurrence risk factors in early-stage cervical cancer based on intratumoral and peritumoral regions, thereby enhancing the value of personalized precision treatment (35). Huang et al. investigated a staging model integrating rFOV diffusion - weighted imaging - derived radiomics with clinical features. In the training cohort, the AUC of the clinical radiomics model (AUC = 0.877) for cervical carcinoma staging was similar to that of the radiomics model (AUC = 0.867) and higher than that of the clinical model (AUC = 0.673) (36). There is also literature on the radiomics of T2WI and T2WI combined with DWI, which suggests that the DWI sequence may provide additional value for pretreatment risk assessment and for guiding personalized treatment strategies in cervical cancer (CC) (37). These studies are consistent with our findings, showing the important role of DWI sequences in the evaluation and prediction of cervical cancer patients.

Our study has several limitations. Firstly, manual ROI delineation involves subjective empirical errors during the sketching process, necessitating subsequent comparison and analysis with automated and semi-automatic segmentation methods. Secondly, the number of cases is limited, as the data were sourced from a single hospital. Subsequent multicenter studies are required to obtain larger samples to further explore this issue. Thirdly, heterogeneity may exist during data acquisition using different scanners and parameters. Although we have employed the Z-score normalization method and utilized ensemble learning models to enhance anti-interference capability, this heterogeneity still poses a potential impact on the stability of radiomic features. To test the model's generalization ability, future studies should include patients from other hospitals while strengthening spatial consistency and device-specific gray-scale correction. Fourthly, a six-month follow-up may not capture long-term trends, therefore future studies should investigate longer follow-up periods. In addition, selecting cases in the locally advanced clinical stage may limit the model's general applicability to other stages of the disease.

## 5 Conclusion

In conclusion, the application value in predicting the clinical outcome of LACC patients who received chemoradiotherapy was compared. This method can non-invasively predict the clinical outcomes of local recurrence or distant metastases. It offers objective and scientific data to support clinical decision-making, enabling clinicians to develop individualized treatment plans in a more rational and scientific manner.

## Data Availability

The original contributions presented in the study are included in the article/**Supplementary Material**. Further inquiries can be directed to the corresponding author.
