# Supplementary material for: Development, validation, and visualization of a novel nomogram for predicting clinical outcomes of radiotherapy combined with chemotherapy in locally advanced cervical cancer
Source: Front Oncol. 2025 Oct 8;15:1668971. doi: 10.3389/fonc.2025.1668971 (PMC12540123; doi:10.3389/fonc.2025.1668971)
Supplement: Supplementary file 1 [file DataSheet1.docx]

**Supplementary material**

**Supplementary Table 1. The detailed MRI scanning protocols**

|  | TR  (ms) | TE  (ms) | field of view  (mm^2^) | matrix | layer thickness  (mm) | Flip angle | Number of averages |
| --- | --- | --- | --- | --- | --- | --- | --- |
| GE | 5000 | 68.1 | 256×256 | 128×160 | 6 | 90 | 4 |
| Siemens | 5400 | 61 | 384×312 | 192×117 | 6 | 90 | 6 |
| Philips | 6000 | 54.9 | 192×192 | 120×143 | 6 | 90 | 2 |


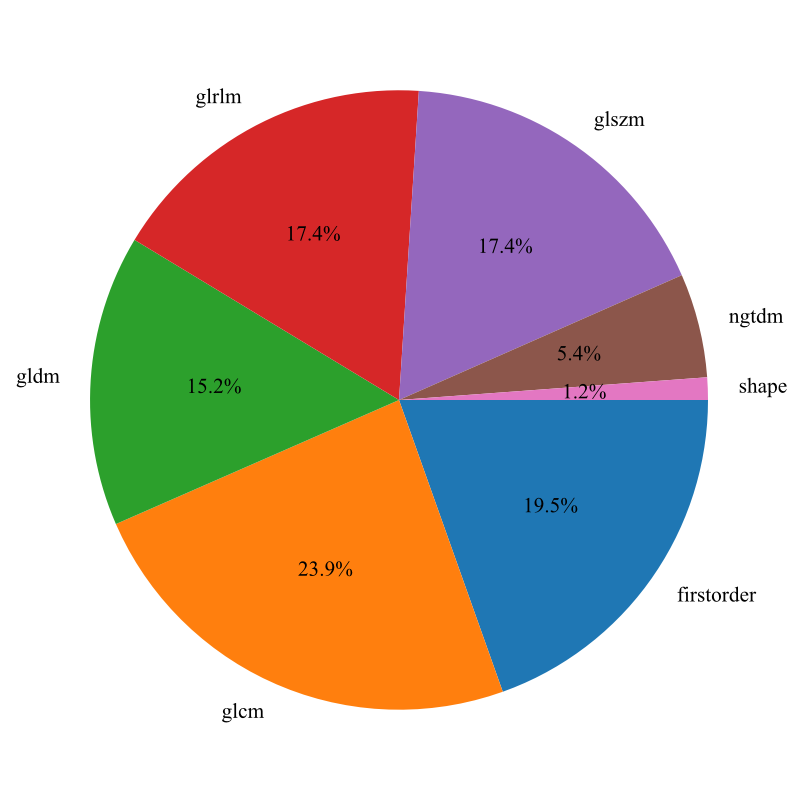


**Supplementary Figure 1. Distribution of radiomics features across different classifications.**


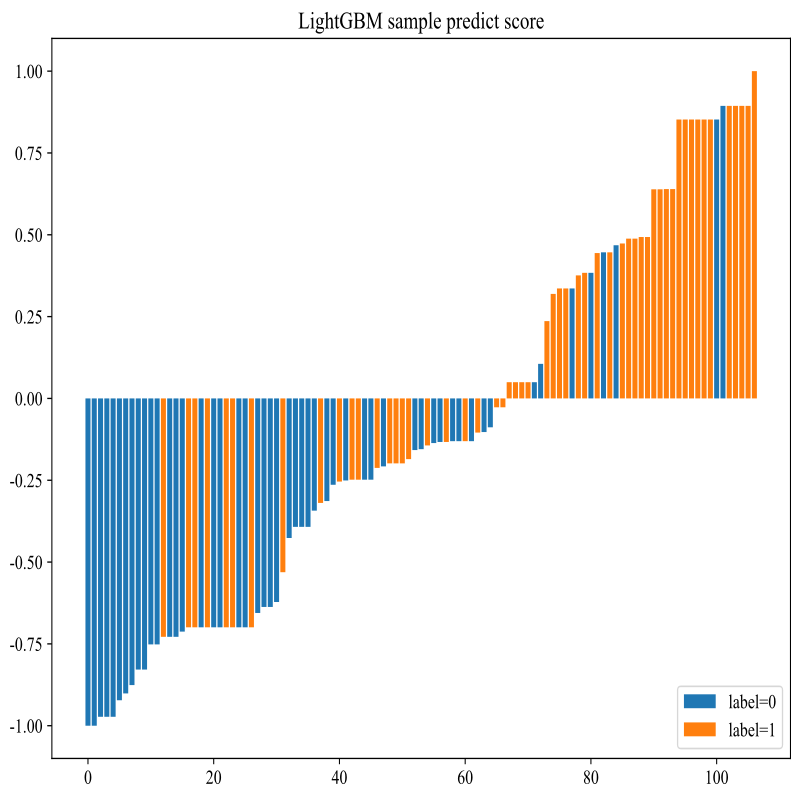

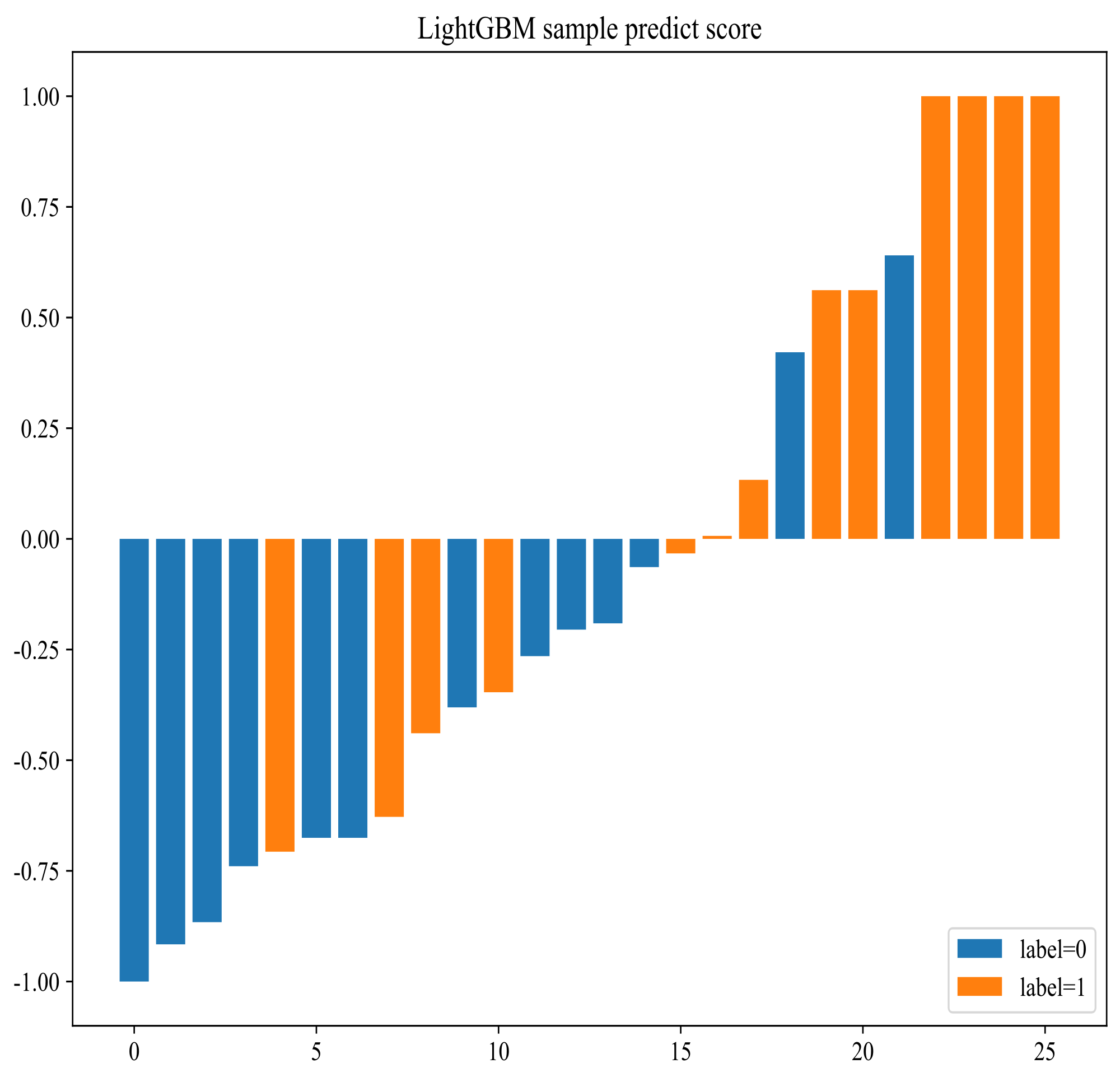


**Supplementary Figure 2. Visualization of sample predictions by LightGBM algorithm in training set and validation set.**

**The extraction of radiomic features**

log_sigma_2_0_mm_3D_firstorder_10Percentile log_sigma_2_0_mm_3D_firstorder_90Percentile log_sigma_2_0_mm_3D_firstorder_Energy log_sigma_2_0_mm_3D_firstorder_Entropy log_sigma_2_0_mm_3D_firstorder_InterquartileRange log_sigma_2_0_mm_3D_firstorder_Kurtosis log_sigma_2_0_mm_3D_firstorder_Maximum log_sigma_2_0_mm_3D_firstorder_Mean log_sigma_2_0_mm_3D_firstorder_MeanAbsoluteDeviation log_sigma_2_0_mm_3D_firstorder_Median log_sigma_2_0_mm_3D_firstorder_Minimum log_sigma_2_0_mm_3D_firstorder_Range log_sigma_2_0_mm_3D_firstorder_RobustMeanAbsoluteDeviation log_sigma_2_0_mm_3D_firstorder_RootMeanSquared log_sigma_2_0_mm_3D_firstorder_Skewness log_sigma_2_0_mm_3D_firstorder_TotalEnergy log_sigma_2_0_mm_3D_firstorder_Uniformity log_sigma_2_0_mm_3D_firstorder_Variance log_sigma_2_0_mm_3D_glcm_Autocorrelation log_sigma_2_0_mm_3D_glcm_ClusterProminence log_sigma_2_0_mm_3D_glcm_ClusterShade log_sigma_2_0_mm_3D_glcm_ClusterTendency log_sigma_2_0_mm_3D_glcm_Contrast log_sigma_2_0_mm_3D_glcm_Correlation log_sigma_2_0_mm_3D_glcm_DifferenceAverage log_sigma_2_0_mm_3D_glcm_DifferenceEntropy log_sigma_2_0_mm_3D_glcm_DifferenceVariance log_sigma_2_0_mm_3D_glcm_Id log_sigma_2_0_mm_3D_glcm_Idm log_sigma_2_0_mm_3D_glcm_Idmn log_sigma_2_0_mm_3D_glcm_Idn log_sigma_2_0_mm_3D_glcm_Imc1 log_sigma_2_0_mm_3D_glcm_Imc2 log_sigma_2_0_mm_3D_glcm_InverseVariance log_sigma_2_0_mm_3D_glcm_JointAverage log_sigma_2_0_mm_3D_glcm_JointEnergy log_sigma_2_0_mm_3D_glcm_JointEntropy log_sigma_2_0_mm_3D_glcm_MaximumProbability log_sigma_2_0_mm_3D_glcm_SumEntropy log_sigma_2_0_mm_3D_glcm_SumSquares log_sigma_2_0_mm_3D_gldm_DependenceEntropy log_sigma_2_0_mm_3D_gldm_DependenceNonUniformity log_sigma_2_0_mm_3D_gldm_DependenceNonUniformityNormalized log_sigma_2_0_mm_3D_gldm_DependenceVariance log_sigma_2_0_mm_3D_gldm_GrayLevelNonUniformity log_sigma_2_0_mm_3D_gldm_GrayLevelVariance log_sigma_2_0_mm_3D_gldm_HighGrayLevelEmphasis log_sigma_2_0_mm_3D_gldm_LargeDependenceEmphasis log_sigma_2_0_mm_3D_gldm_LargeDependenceHighGrayLevelEmphasis log_sigma_2_0_mm_3D_gldm_LargeDependenceLowGrayLevelEmphasis log_sigma_2_0_mm_3D_gldm_LowGrayLevelEmphasis log_sigma_2_0_mm_3D_gldm_SmallDependenceEmphasis log_sigma_2_0_mm_3D_gldm_SmallDependenceHighGrayLevelEmphasis log_sigma_2_0_mm_3D_gldm_SmallDependenceLowGrayLevelEmphasis log_sigma_2_0_mm_3D_glrlm_GrayLevelNonUniformity log_sigma_2_0_mm_3D_glrlm_GrayLevelNonUniformityNormalized log_sigma_2_0_mm_3D_glrlm_GrayLevelVariance log_sigma_2_0_mm_3D_glrlm_HighGrayLevelRunEmphasis log_sigma_2_0_mm_3D_glrlm_LongRunEmphasis log_sigma_2_0_mm_3D_glrlm_LongRunHighGrayLevelEmphasis log_sigma_2_0_mm_3D_glrlm_LongRunLowGrayLevelEmphasis log_sigma_2_0_mm_3D_glrlm_LowGrayLevelRunEmphasis log_sigma_2_0_mm_3D_glrlm_RunEntropy log_sigma_2_0_mm_3D_glrlm_RunLengthNonUniformity log_sigma_2_0_mm_3D_glrlm_RunLengthNonUniformityNormalized log_sigma_2_0_mm_3D_glrlm_RunPercentage log_sigma_2_0_mm_3D_glrlm_RunVariance log_sigma_2_0_mm_3D_glrlm_ShortRunEmphasis log_sigma_2_0_mm_3D_glrlm_ShortRunHighGrayLevelEmphasis log_sigma_2_0_mm_3D_glrlm_ShortRunLowGrayLevelEmphasis log_sigma_2_0_mm_3D_glszm_GrayLevelNonUniformity log_sigma_2_0_mm_3D_glszm_GrayLevelNonUniformityNormalized log_sigma_2_0_mm_3D_glszm_GrayLevelVariance log_sigma_2_0_mm_3D_glszm_HighGrayLevelZoneEmphasis log_sigma_2_0_mm_3D_glszm_LargeAreaEmphasis log_sigma_2_0_mm_3D_glszm_LargeAreaHighGrayLevelEmphasis log_sigma_2_0_mm_3D_glszm_LargeAreaLowGrayLevelEmphasis log_sigma_2_0_mm_3D_glszm_LowGrayLevelZoneEmphasis log_sigma_2_0_mm_3D_glszm_SizeZoneNonUniformity log_sigma_2_0_mm_3D_glszm_SizeZoneNonUniformityNormalized log_sigma_2_0_mm_3D_glszm_SmallAreaEmphasis log_sigma_2_0_mm_3D_glszm_SmallAreaHighGrayLevelEmphasis log_sigma_2_0_mm_3D_glszm_SmallAreaLowGrayLevelEmphasis log_sigma_2_0_mm_3D_glszm_ZoneEntropy log_sigma_2_0_mm_3D_glszm_ZonePercentage log_sigma_2_0_mm_3D_glszm_ZoneVariance log_sigma_2_0_mm_3D_ngtdm_Busyness log_sigma_2_0_mm_3D_ngtdm_Coarseness log_sigma_2_0_mm_3D_ngtdm_Complexity log_sigma_2_0_mm_3D_ngtdm_Contrast log_sigma_2_0_mm_3D_ngtdm_Strength log_sigma_3_0_mm_3D_firstorder_10Percentile log_sigma_3_0_mm_3D_firstorder_90Percentile log_sigma_3_0_mm_3D_firstorder_Energy log_sigma_3_0_mm_3D_firstorder_Entropy log_sigma_3_0_mm_3D_firstorder_InterquartileRange log_sigma_3_0_mm_3D_firstorder_Kurtosis log_sigma_3_0_mm_3D_firstorder_Maximum log_sigma_3_0_mm_3D_firstorder_Mean log_sigma_3_0_mm_3D_firstorder_MeanAbsoluteDeviation log_sigma_3_0_mm_3D_firstorder_Median log_sigma_3_0_mm_3D_firstorder_Minimum log_sigma_3_0_mm_3D_firstorder_Range log_sigma_3_0_mm_3D_firstorder_RobustMeanAbsoluteDeviation log_sigma_3_0_mm_3D_firstorder_RootMeanSquared log_sigma_3_0_mm_3D_firstorder_Skewness log_sigma_3_0_mm_3D_firstorder_TotalEnergy log_sigma_3_0_mm_3D_firstorder_Uniformity log_sigma_3_0_mm_3D_firstorder_Variance log_sigma_3_0_mm_3D_glcm_Autocorrelation log_sigma_3_0_mm_3D_glcm_ClusterProminence log_sigma_3_0_mm_3D_glcm_ClusterShade log_sigma_3_0_mm_3D_glcm_ClusterTendency log_sigma_3_0_mm_3D_glcm_Contrast log_sigma_3_0_mm_3D_glcm_Correlation log_sigma_3_0_mm_3D_glcm_DifferenceAverage log_sigma_3_0_mm_3D_glcm_DifferenceEntropy log_sigma_3_0_mm_3D_glcm_DifferenceVariance log_sigma_3_0_mm_3D_glcm_Id log_sigma_3_0_mm_3D_glcm_Idm log_sigma_3_0_mm_3D_glcm_Idmn log_sigma_3_0_mm_3D_glcm_Idn log_sigma_3_0_mm_3D_glcm_Imc1 log_sigma_3_0_mm_3D_glcm_Imc2 log_sigma_3_0_mm_3D_glcm_InverseVariance log_sigma_3_0_mm_3D_glcm_JointAverage log_sigma_3_0_mm_3D_glcm_JointEnergy log_sigma_3_0_mm_3D_glcm_JointEntropy log_sigma_3_0_mm_3D_glcm_MaximumProbability log_sigma_3_0_mm_3D_glcm_SumEntropy log_sigma_3_0_mm_3D_glcm_SumSquares log_sigma_3_0_mm_3D_gldm_DependenceEntropy log_sigma_3_0_mm_3D_gldm_DependenceNonUniformity log_sigma_3_0_mm_3D_gldm_DependenceNonUniformityNormalized log_sigma_3_0_mm_3D_gldm_DependenceVariance log_sigma_3_0_mm_3D_gldm_GrayLevelNonUniformity log_sigma_3_0_mm_3D_gldm_GrayLevelVariance log_sigma_3_0_mm_3D_gldm_HighGrayLevelEmphasis log_sigma_3_0_mm_3D_gldm_LargeDependenceEmphasis log_sigma_3_0_mm_3D_gldm_LargeDependenceHighGrayLevelEmphasis log_sigma_3_0_mm_3D_gldm_LargeDependenceLowGrayLevelEmphasis log_sigma_3_0_mm_3D_gldm_LowGrayLevelEmphasis log_sigma_3_0_mm_3D_gldm_SmallDependenceEmphasis log_sigma_3_0_mm_3D_gldm_SmallDependenceHighGrayLevelEmphasis log_sigma_3_0_mm_3D_gldm_SmallDependenceLowGrayLevelEmphasis log_sigma_3_0_mm_3D_glrlm_GrayLevelNonUniformity log_sigma_3_0_mm_3D_glrlm_GrayLevelNonUniformityNormalized log_sigma_3_0_mm_3D_glrlm_GrayLevelVariance log_sigma_3_0_mm_3D_glrlm_HighGrayLevelRunEmphasis log_sigma_3_0_mm_3D_glrlm_LongRunEmphasis log_sigma_3_0_mm_3D_glrlm_LongRunHighGrayLevelEmphasis log_sigma_3_0_mm_3D_glrlm_LongRunLowGrayLevelEmphasis log_sigma_3_0_mm_3D_glrlm_LowGrayLevelRunEmphasis log_sigma_3_0_mm_3D_glrlm_RunEntropy log_sigma_3_0_mm_3D_glrlm_RunLengthNonUniformity log_sigma_3_0_mm_3D_glrlm_RunLengthNonUniformityNormalized log_sigma_3_0_mm_3D_glrlm_RunPercentage log_sigma_3_0_mm_3D_glrlm_RunVariance log_sigma_3_0_mm_3D_glrlm_ShortRunEmphasis log_sigma_3_0_mm_3D_glrlm_ShortRunHighGrayLevelEmphasis log_sigma_3_0_mm_3D_glrlm_ShortRunLowGrayLevelEmphasis log_sigma_3_0_mm_3D_glszm_GrayLevelNonUniformity log_sigma_3_0_mm_3D_glszm_GrayLevelNonUniformityNormalized log_sigma_3_0_mm_3D_glszm_GrayLevelVariance log_sigma_3_0_mm_3D_glszm_HighGrayLevelZoneEmphasis log_sigma_3_0_mm_3D_glszm_LargeAreaEmphasis log_sigma_3_0_mm_3D_glszm_LargeAreaHighGrayLevelEmphasis log_sigma_3_0_mm_3D_glszm_LargeAreaLowGrayLevelEmphasis log_sigma_3_0_mm_3D_glszm_LowGrayLevelZoneEmphasis log_sigma_3_0_mm_3D_glszm_SizeZoneNonUniformity log_sigma_3_0_mm_3D_glszm_SizeZoneNonUniformityNormalized log_sigma_3_0_mm_3D_glszm_SmallAreaEmphasis log_sigma_3_0_mm_3D_glszm_SmallAreaHighGrayLevelEmphasis log_sigma_3_0_mm_3D_glszm_SmallAreaLowGrayLevelEmphasis log_sigma_3_0_mm_3D_glszm_ZoneEntropy log_sigma_3_0_mm_3D_glszm_ZonePercentage log_sigma_3_0_mm_3D_glszm_ZoneVariance log_sigma_3_0_mm_3D_ngtdm_Busyness log_sigma_3_0_mm_3D_ngtdm_Coarseness log_sigma_3_0_mm_3D_ngtdm_Complexity log_sigma_3_0_mm_3D_ngtdm_Contrast log_sigma_3_0_mm_3D_ngtdm_Strength log_sigma_4_0_mm_3D_firstorder_10Percentile log_sigma_4_0_mm_3D_firstorder_90Percentile log_sigma_4_0_mm_3D_firstorder_Energy log_sigma_4_0_mm_3D_firstorder_Entropy log_sigma_4_0_mm_3D_firstorder_InterquartileRange log_sigma_4_0_mm_3D_firstorder_Kurtosis log_sigma_4_0_mm_3D_firstorder_Maximum log_sigma_4_0_mm_3D_firstorder_Mean log_sigma_4_0_mm_3D_firstorder_MeanAbsoluteDeviation log_sigma_4_0_mm_3D_firstorder_Median log_sigma_4_0_mm_3D_firstorder_Minimum log_sigma_4_0_mm_3D_firstorder_Range log_sigma_4_0_mm_3D_firstorder_RobustMeanAbsoluteDeviation log_sigma_4_0_mm_3D_firstorder_RootMeanSquared log_sigma_4_0_mm_3D_firstorder_Skewness log_sigma_4_0_mm_3D_firstorder_TotalEnergy log_sigma_4_0_mm_3D_firstorder_Uniformity log_sigma_4_0_mm_3D_firstorder_Variance log_sigma_4_0_mm_3D_glcm_Autocorrelation log_sigma_4_0_mm_3D_glcm_ClusterProminence log_sigma_4_0_mm_3D_glcm_ClusterShade log_sigma_4_0_mm_3D_glcm_ClusterTendency log_sigma_4_0_mm_3D_glcm_Contrast log_sigma_4_0_mm_3D_glcm_Correlation log_sigma_4_0_mm_3D_glcm_DifferenceAverage log_sigma_4_0_mm_3D_glcm_DifferenceEntropy log_sigma_4_0_mm_3D_glcm_DifferenceVariance log_sigma_4_0_mm_3D_glcm_Id log_sigma_4_0_mm_3D_glcm_Idm log_sigma_4_0_mm_3D_glcm_Idmn log_sigma_4_0_mm_3D_glcm_Idn log_sigma_4_0_mm_3D_glcm_Imc1 log_sigma_4_0_mm_3D_glcm_Imc2 log_sigma_4_0_mm_3D_glcm_InverseVariance log_sigma_4_0_mm_3D_glcm_JointAverage log_sigma_4_0_mm_3D_glcm_JointEnergy log_sigma_4_0_mm_3D_glcm_JointEntropy log_sigma_4_0_mm_3D_glcm_MaximumProbability log_sigma_4_0_mm_3D_glcm_SumEntropy log_sigma_4_0_mm_3D_glcm_SumSquares log_sigma_4_0_mm_3D_gldm_DependenceEntropy log_sigma_4_0_mm_3D_gldm_DependenceNonUniformity log_sigma_4_0_mm_3D_gldm_DependenceNonUniformityNormalized log_sigma_4_0_mm_3D_gldm_DependenceVariance log_sigma_4_0_mm_3D_gldm_GrayLevelNonUniformity log_sigma_4_0_mm_3D_gldm_GrayLevelVariance log_sigma_4_0_mm_3D_gldm_HighGrayLevelEmphasis log_sigma_4_0_mm_3D_gldm_LargeDependenceEmphasis log_sigma_4_0_mm_3D_gldm_LargeDependenceHighGrayLevelEmphasis log_sigma_4_0_mm_3D_gldm_LargeDependenceLowGrayLevelEmphasis log_sigma_4_0_mm_3D_gldm_LowGrayLevelEmphasis log_sigma_4_0_mm_3D_gldm_SmallDependenceEmphasis log_sigma_4_0_mm_3D_gldm_SmallDependenceHighGrayLevelEmphasis log_sigma_4_0_mm_3D_gldm_SmallDependenceLowGrayLevelEmphasis log_sigma_4_0_mm_3D_glrlm_GrayLevelNonUniformity log_sigma_4_0_mm_3D_glrlm_GrayLevelNonUniformityNormalized log_sigma_4_0_mm_3D_glrlm_GrayLevelVariance log_sigma_4_0_mm_3D_glrlm_HighGrayLevelRunEmphasis log_sigma_4_0_mm_3D_glrlm_LongRunEmphasis log_sigma_4_0_mm_3D_glrlm_LongRunHighGrayLevelEmphasis log_sigma_4_0_mm_3D_glrlm_LongRunLowGrayLevelEmphasis log_sigma_4_0_mm_3D_glrlm_LowGrayLevelRunEmphasis log_sigma_4_0_mm_3D_glrlm_RunEntropy log_sigma_4_0_mm_3D_glrlm_RunLengthNonUniformity log_sigma_4_0_mm_3D_glrlm_RunLengthNonUniformityNormalized log_sigma_4_0_mm_3D_glrlm_RunPercentage log_sigma_4_0_mm_3D_glrlm_RunVariance log_sigma_4_0_mm_3D_glrlm_ShortRunEmphasis log_sigma_4_0_mm_3D_glrlm_ShortRunHighGrayLevelEmphasis log_sigma_4_0_mm_3D_glrlm_ShortRunLowGrayLevelEmphasis log_sigma_4_0_mm_3D_glszm_GrayLevelNonUniformity log_sigma_4_0_mm_3D_glszm_GrayLevelNonUniformityNormalized log_sigma_4_0_mm_3D_glszm_GrayLevelVariance log_sigma_4_0_mm_3D_glszm_HighGrayLevelZoneEmphasis log_sigma_4_0_mm_3D_glszm_LargeAreaEmphasis log_sigma_4_0_mm_3D_glszm_LargeAreaHighGrayLevelEmphasis log_sigma_4_0_mm_3D_glszm_LargeAreaLowGrayLevelEmphasis log_sigma_4_0_mm_3D_glszm_LowGrayLevelZoneEmphasis log_sigma_4_0_mm_3D_glszm_SizeZoneNonUniformity log_sigma_4_0_mm_3D_glszm_SizeZoneNonUniformityNormalized log_sigma_4_0_mm_3D_glszm_SmallAreaEmphasis log_sigma_4_0_mm_3D_glszm_SmallAreaHighGrayLevelEmphasis log_sigma_4_0_mm_3D_glszm_SmallAreaLowGrayLevelEmphasis log_sigma_4_0_mm_3D_glszm_ZoneEntropy log_sigma_4_0_mm_3D_glszm_ZonePercentage log_sigma_4_0_mm_3D_glszm_ZoneVariance log_sigma_4_0_mm_3D_ngtdm_Busyness log_sigma_4_0_mm_3D_ngtdm_Coarseness log_sigma_4_0_mm_3D_ngtdm_Complexity log_sigma_4_0_mm_3D_ngtdm_Contrast log_sigma_4_0_mm_3D_ngtdm_Strength log_sigma_5_0_mm_3D_firstorder_10Percentile log_sigma_5_0_mm_3D_firstorder_90Percentile log_sigma_5_0_mm_3D_firstorder_Energy log_sigma_5_0_mm_3D_firstorder_Entropy log_sigma_5_0_mm_3D_firstorder_InterquartileRange log_sigma_5_0_mm_3D_firstorder_Kurtosis log_sigma_5_0_mm_3D_firstorder_Maximum log_sigma_5_0_mm_3D_firstorder_Mean log_sigma_5_0_mm_3D_firstorder_MeanAbsoluteDeviation log_sigma_5_0_mm_3D_firstorder_Median log_sigma_5_0_mm_3D_firstorder_Minimum log_sigma_5_0_mm_3D_firstorder_Range log_sigma_5_0_mm_3D_firstorder_RobustMeanAbsoluteDeviation log_sigma_5_0_mm_3D_firstorder_RootMeanSquared log_sigma_5_0_mm_3D_firstorder_Skewness log_sigma_5_0_mm_3D_firstorder_TotalEnergy log_sigma_5_0_mm_3D_firstorder_Uniformity log_sigma_5_0_mm_3D_firstorder_Variance log_sigma_5_0_mm_3D_glcm_Autocorrelation log_sigma_5_0_mm_3D_glcm_ClusterProminence log_sigma_5_0_mm_3D_glcm_ClusterShade log_sigma_5_0_mm_3D_glcm_ClusterTendency log_sigma_5_0_mm_3D_glcm_Contrast log_sigma_5_0_mm_3D_glcm_Correlation log_sigma_5_0_mm_3D_glcm_DifferenceAverage log_sigma_5_0_mm_3D_glcm_DifferenceEntropy log_sigma_5_0_mm_3D_glcm_DifferenceVariance log_sigma_5_0_mm_3D_glcm_Id log_sigma_5_0_mm_3D_glcm_Idm log_sigma_5_0_mm_3D_glcm_Idmn log_sigma_5_0_mm_3D_glcm_Idn log_sigma_5_0_mm_3D_glcm_Imc1 log_sigma_5_0_mm_3D_glcm_Imc2 log_sigma_5_0_mm_3D_glcm_InverseVariance log_sigma_5_0_mm_3D_glcm_JointAverage log_sigma_5_0_mm_3D_glcm_JointEnergy log_sigma_5_0_mm_3D_glcm_JointEntropy log_sigma_5_0_mm_3D_glcm_MaximumProbability log_sigma_5_0_mm_3D_glcm_SumEntropy log_sigma_5_0_mm_3D_glcm_SumSquares log_sigma_5_0_mm_3D_gldm_DependenceEntropy log_sigma_5_0_mm_3D_gldm_DependenceNonUniformity log_sigma_5_0_mm_3D_gldm_DependenceNonUniformityNormalized log_sigma_5_0_mm_3D_gldm_DependenceVariance log_sigma_5_0_mm_3D_gldm_GrayLevelNonUniformity log_sigma_5_0_mm_3D_gldm_GrayLevelVariance log_sigma_5_0_mm_3D_gldm_HighGrayLevelEmphasis log_sigma_5_0_mm_3D_gldm_LargeDependenceEmphasis log_sigma_5_0_mm_3D_gldm_LargeDependenceHighGrayLevelEmphasis log_sigma_5_0_mm_3D_gldm_LargeDependenceLowGrayLevelEmphasis log_sigma_5_0_mm_3D_gldm_LowGrayLevelEmphasis log_sigma_5_0_mm_3D_gldm_SmallDependenceEmphasis log_sigma_5_0_mm_3D_gldm_SmallDependenceHighGrayLevelEmphasis log_sigma_5_0_mm_3D_gldm_SmallDependenceLowGrayLevelEmphasis log_sigma_5_0_mm_3D_glrlm_GrayLevelNonUniformity log_sigma_5_0_mm_3D_glrlm_GrayLevelNonUniformityNormalized log_sigma_5_0_mm_3D_glrlm_GrayLevelVariance log_sigma_5_0_mm_3D_glrlm_HighGrayLevelRunEmphasis log_sigma_5_0_mm_3D_glrlm_LongRunEmphasis log_sigma_5_0_mm_3D_glrlm_LongRunHighGrayLevelEmphasis log_sigma_5_0_mm_3D_glrlm_LongRunLowGrayLevelEmphasis log_sigma_5_0_mm_3D_glrlm_LowGrayLevelRunEmphasis log_sigma_5_0_mm_3D_glrlm_RunEntropy log_sigma_5_0_mm_3D_glrlm_RunLengthNonUniformity log_sigma_5_0_mm_3D_glrlm_RunLengthNonUniformityNormalized log_sigma_5_0_mm_3D_glrlm_RunPercentage log_sigma_5_0_mm_3D_glrlm_RunVariance log_sigma_5_0_mm_3D_glrlm_ShortRunEmphasis log_sigma_5_0_mm_3D_glrlm_ShortRunHighGrayLevelEmphasis log_sigma_5_0_mm_3D_glrlm_ShortRunLowGrayLevelEmphasis log_sigma_5_0_mm_3D_glszm_GrayLevelNonUniformity log_sigma_5_0_mm_3D_glszm_GrayLevelNonUniformityNormalized log_sigma_5_0_mm_3D_glszm_GrayLevelVariance log_sigma_5_0_mm_3D_glszm_HighGrayLevelZoneEmphasis log_sigma_5_0_mm_3D_glszm_LargeAreaEmphasis log_sigma_5_0_mm_3D_glszm_LargeAreaHighGrayLevelEmphasis log_sigma_5_0_mm_3D_glszm_LargeAreaLowGrayLevelEmphasis log_sigma_5_0_mm_3D_glszm_LowGrayLevelZoneEmphasis log_sigma_5_0_mm_3D_glszm_SizeZoneNonUniformity log_sigma_5_0_mm_3D_glszm_SizeZoneNonUniformityNormalized log_sigma_5_0_mm_3D_glszm_SmallAreaEmphasis log_sigma_5_0_mm_3D_glszm_SmallAreaHighGrayLevelEmphasis log_sigma_5_0_mm_3D_glszm_SmallAreaLowGrayLevelEmphasis log_sigma_5_0_mm_3D_glszm_ZoneEntropy log_sigma_5_0_mm_3D_glszm_ZonePercentage log_sigma_5_0_mm_3D_glszm_ZoneVariance log_sigma_5_0_mm_3D_ngtdm_Busyness log_sigma_5_0_mm_3D_ngtdm_Coarseness log_sigma_5_0_mm_3D_ngtdm_Complexity log_sigma_5_0_mm_3D_ngtdm_Contrast log_sigma_5_0_mm_3D_ngtdm_Strength original_firstorder_10Percentile original_firstorder_90Percentile original_firstorder_Energy original_firstorder_Entropy original_firstorder_InterquartileRange original_firstorder_Kurtosis original_firstorder_Maximum original_firstorder_Mean original_firstorder_MeanAbsoluteDeviation original_firstorder_Median original_firstorder_Minimum original_firstorder_Range original_firstorder_RobustMeanAbsoluteDeviation original_firstorder_RootMeanSquared original_firstorder_Skewness original_firstorder_TotalEnergy original_firstorder_Uniformity original_firstorder_Variance original_glcm_Autocorrelation original_glcm_ClusterProminence original_glcm_ClusterShade original_glcm_ClusterTendency original_glcm_Contrast original_glcm_Correlation original_glcm_DifferenceAverage original_glcm_DifferenceEntropy original_glcm_DifferenceVariance original_glcm_Id original_glcm_Idm original_glcm_Idmn original_glcm_Idn original_glcm_Imc1 original_glcm_Imc2 original_glcm_InverseVariance original_glcm_JointAverage original_glcm_JointEnergy original_glcm_JointEntropy original_glcm_MaximumProbability original_glcm_SumEntropy original_glcm_SumSquares original_gldm_DependenceEntropy original_gldm_DependenceNonUniformity original_gldm_DependenceNonUniformityNormalized original_gldm_DependenceVariance original_gldm_GrayLevelNonUniformity original_gldm_GrayLevelVariance original_gldm_HighGrayLevelEmphasis original_gldm_LargeDependenceEmphasis original_gldm_LargeDependenceHighGrayLevelEmphasis original_gldm_LargeDependenceLowGrayLevelEmphasis original_gldm_LowGrayLevelEmphasis original_gldm_SmallDependenceEmphasis original_gldm_SmallDependenceHighGrayLevelEmphasis original_gldm_SmallDependenceLowGrayLevelEmphasis original_glrlm_GrayLevelNonUniformity original_glrlm_GrayLevelNonUniformityNormalized original_glrlm_GrayLevelVariance original_glrlm_HighGrayLevelRunEmphasis original_glrlm_LongRunEmphasis original_glrlm_LongRunHighGrayLevelEmphasis original_glrlm_LongRunLowGrayLevelEmphasis original_glrlm_LowGrayLevelRunEmphasis original_glrlm_RunEntropy original_glrlm_RunLengthNonUniformity original_glrlm_RunLengthNonUniformityNormalized original_glrlm_RunPercentage original_glrlm_RunVariance original_glrlm_ShortRunEmphasis original_glrlm_ShortRunHighGrayLevelEmphasis original_glrlm_ShortRunLowGrayLevelEmphasis original_glszm_GrayLevelNonUniformity original_glszm_GrayLevelNonUniformityNormalized original_glszm_GrayLevelVariance original_glszm_HighGrayLevelZoneEmphasis original_glszm_LargeAreaEmphasis original_glszm_LargeAreaHighGrayLevelEmphasis original_glszm_LargeAreaLowGrayLevelEmphasis original_glszm_LowGrayLevelZoneEmphasis original_glszm_SizeZoneNonUniformity original_glszm_SizeZoneNonUniformityNormalized original_glszm_SmallAreaEmphasis original_glszm_SmallAreaHighGrayLevelEmphasis original_glszm_SmallAreaLowGrayLevelEmphasis original_glszm_ZoneEntropy original_glszm_ZonePercentage original_glszm_ZoneVariance original_ngtdm_Busyness original_ngtdm_Coarseness original_ngtdm_Complexity original_ngtdm_Contrast original_ngtdm_Strength original_shape_Elongation original_shape_Flatness original_shape_LeastAxisLength original_shape_MajorAxisLength original_shape_Maximum2DDiameterColumn original_shape_Maximum2DDiameterRow original_shape_Maximum2DDiameterSlice original_shape_Maximum3DDiameter original_shape_MeshVolume original_shape_MinorAxisLength original_shape_Sphericity original_shape_SurfaceArea original_shape_SurfaceVolumeRatio original_shape_VoxelVolume wavelet_HHH_firstorder_10Percentile wavelet_HHH_firstorder_90Percentile wavelet_HHH_firstorder_Energy wavelet_HHH_firstorder_Entropy wavelet_HHH_firstorder_InterquartileRange wavelet_HHH_firstorder_Kurtosis wavelet_HHH_firstorder_Maximum wavelet_HHH_firstorder_Mean wavelet_HHH_firstorder_MeanAbsoluteDeviation wavelet_HHH_firstorder_Median wavelet_HHH_firstorder_Minimum wavelet_HHH_firstorder_Range wavelet_HHH_firstorder_RobustMeanAbsoluteDeviation wavelet_HHH_firstorder_RootMeanSquared wavelet_HHH_firstorder_Skewness wavelet_HHH_firstorder_TotalEnergy wavelet_HHH_firstorder_Uniformity wavelet_HHH_firstorder_Variance wavelet_HHH_glcm_Autocorrelation wavelet_HHH_glcm_ClusterProminence wavelet_HHH_glcm_ClusterShade wavelet_HHH_glcm_ClusterTendency wavelet_HHH_glcm_Contrast wavelet_HHH_glcm_Correlation wavelet_HHH_glcm_DifferenceAverage wavelet_HHH_glcm_DifferenceEntropy wavelet_HHH_glcm_DifferenceVariance wavelet_HHH_glcm_Id wavelet_HHH_glcm_Idm wavelet_HHH_glcm_Idmn wavelet_HHH_glcm_Idn wavelet_HHH_glcm_Imc1 wavelet_HHH_glcm_Imc2 wavelet_HHH_glcm_InverseVariance wavelet_HHH_glcm_JointAverage wavelet_HHH_glcm_JointEnergy wavelet_HHH_glcm_JointEntropy wavelet_HHH_glcm_MaximumProbability wavelet_HHH_glcm_SumEntropy wavelet_HHH_glcm_SumSquares wavelet_HHH_gldm_DependenceEntropy wavelet_HHH_gldm_DependenceNonUniformity wavelet_HHH_gldm_DependenceNonUniformityNormalized wavelet_HHH_gldm_DependenceVariance wavelet_HHH_gldm_GrayLevelNonUniformity wavelet_HHH_gldm_GrayLevelVariance wavelet_HHH_gldm_HighGrayLevelEmphasis wavelet_HHH_gldm_LargeDependenceEmphasis wavelet_HHH_gldm_LargeDependenceHighGrayLevelEmphasis wavelet_HHH_gldm_LargeDependenceLowGrayLevelEmphasis wavelet_HHH_gldm_LowGrayLevelEmphasis wavelet_HHH_gldm_SmallDependenceEmphasis wavelet_HHH_gldm_SmallDependenceHighGrayLevelEmphasis wavelet_HHH_gldm_SmallDependenceLowGrayLevelEmphasis wavelet_HHH_glrlm_GrayLevelNonUniformity wavelet_HHH_glrlm_GrayLevelNonUniformityNormalized wavelet_HHH_glrlm_GrayLevelVariance wavelet_HHH_glrlm_HighGrayLevelRunEmphasis wavelet_HHH_glrlm_LongRunEmphasis wavelet_HHH_glrlm_LongRunHighGrayLevelEmphasis wavelet_HHH_glrlm_LongRunLowGrayLevelEmphasis wavelet_HHH_glrlm_LowGrayLevelRunEmphasis wavelet_HHH_glrlm_RunEntropy wavelet_HHH_glrlm_RunLengthNonUniformity wavelet_HHH_glrlm_RunLengthNonUniformityNormalized wavelet_HHH_glrlm_RunPercentage wavelet_HHH_glrlm_RunVariance wavelet_HHH_glrlm_ShortRunEmphasis wavelet_HHH_glrlm_ShortRunHighGrayLevelEmphasis wavelet_HHH_glrlm_ShortRunLowGrayLevelEmphasis wavelet_HHH_glszm_GrayLevelNonUniformity wavelet_HHH_glszm_GrayLevelNonUniformityNormalized wavelet_HHH_glszm_GrayLevelVariance wavelet_HHH_glszm_HighGrayLevelZoneEmphasis wavelet_HHH_glszm_LargeAreaEmphasis wavelet_HHH_glszm_LargeAreaHighGrayLevelEmphasis wavelet_HHH_glszm_LargeAreaLowGrayLevelEmphasis wavelet_HHH_glszm_LowGrayLevelZoneEmphasis wavelet_HHH_glszm_SizeZoneNonUniformity wavelet_HHH_glszm_SizeZoneNonUniformityNormalized wavelet_HHH_glszm_SmallAreaEmphasis wavelet_HHH_glszm_SmallAreaHighGrayLevelEmphasis wavelet_HHH_glszm_SmallAreaLowGrayLevelEmphasis wavelet_HHH_glszm_ZoneEntropy wavelet_HHH_glszm_ZonePercentage wavelet_HHH_glszm_ZoneVariance wavelet_HHH_ngtdm_Busyness wavelet_HHH_ngtdm_Coarseness wavelet_HHH_ngtdm_Complexity wavelet_HHH_ngtdm_Contrast wavelet_HHH_ngtdm_Strength wavelet_HHL_firstorder_10Percentile wavelet_HHL_firstorder_90Percentile wavelet_HHL_firstorder_Energy wavelet_HHL_firstorder_Entropy wavelet_HHL_firstorder_InterquartileRange wavelet_HHL_firstorder_Kurtosis wavelet_HHL_firstorder_Maximum wavelet_HHL_firstorder_Mean wavelet_HHL_firstorder_MeanAbsoluteDeviation wavelet_HHL_firstorder_Median wavelet_HHL_firstorder_Minimum wavelet_HHL_firstorder_Range wavelet_HHL_firstorder_RobustMeanAbsoluteDeviation wavelet_HHL_firstorder_RootMeanSquared wavelet_HHL_firstorder_Skewness wavelet_HHL_firstorder_TotalEnergy wavelet_HHL_firstorder_Uniformity wavelet_HHL_firstorder_Variance wavelet_HHL_glcm_Autocorrelation wavelet_HHL_glcm_ClusterProminence wavelet_HHL_glcm_ClusterShade wavelet_HHL_glcm_ClusterTendency wavelet_HHL_glcm_Contrast wavelet_HHL_glcm_Correlation wavelet_HHL_glcm_DifferenceAverage wavelet_HHL_glcm_DifferenceEntropy wavelet_HHL_glcm_DifferenceVariance wavelet_HHL_glcm_Id wavelet_HHL_glcm_Idm wavelet_HHL_glcm_Idmn wavelet_HHL_glcm_Idn wavelet_HHL_glcm_Imc1 wavelet_HHL_glcm_Imc2 wavelet_HHL_glcm_InverseVariance wavelet_HHL_glcm_JointAverage wavelet_HHL_glcm_JointEnergy wavelet_HHL_glcm_JointEntropy wavelet_HHL_glcm_MaximumProbability wavelet_HHL_glcm_SumEntropy wavelet_HHL_glcm_SumSquares wavelet_HHL_gldm_DependenceEntropy wavelet_HHL_gldm_DependenceNonUniformity wavelet_HHL_gldm_DependenceNonUniformityNormalized wavelet_HHL_gldm_DependenceVariance wavelet_HHL_gldm_GrayLevelNonUniformity wavelet_HHL_gldm_GrayLevelVariance wavelet_HHL_gldm_HighGrayLevelEmphasis wavelet_HHL_gldm_LargeDependenceEmphasis wavelet_HHL_gldm_LargeDependenceHighGrayLevelEmphasis wavelet_HHL_gldm_LargeDependenceLowGrayLevelEmphasis wavelet_HHL_gldm_LowGrayLevelEmphasis wavelet_HHL_gldm_SmallDependenceEmphasis wavelet_HHL_gldm_SmallDependenceHighGrayLevelEmphasis wavelet_HHL_gldm_SmallDependenceLowGrayLevelEmphasis wavelet_HHL_glrlm_GrayLevelNonUniformity wavelet_HHL_glrlm_GrayLevelNonUniformityNormalized wavelet_HHL_glrlm_GrayLevelVariance wavelet_HHL_glrlm_HighGrayLevelRunEmphasis wavelet_HHL_glrlm_LongRunEmphasis wavelet_HHL_glrlm_LongRunHighGrayLevelEmphasis wavelet_HHL_glrlm_LongRunLowGrayLevelEmphasis wavelet_HHL_glrlm_LowGrayLevelRunEmphasis wavelet_HHL_glrlm_RunEntropy wavelet_HHL_glrlm_RunLengthNonUniformity wavelet_HHL_glrlm_RunLengthNonUniformityNormalized wavelet_HHL_glrlm_RunPercentage wavelet_HHL_glrlm_RunVariance wavelet_HHL_glrlm_ShortRunEmphasis wavelet_HHL_glrlm_ShortRunHighGrayLevelEmphasis wavelet_HHL_glrlm_ShortRunLowGrayLevelEmphasis wavelet_HHL_glszm_GrayLevelNonUniformity wavelet_HHL_glszm_GrayLevelNonUniformityNormalized wavelet_HHL_glszm_GrayLevelVariance wavelet_HHL_glszm_HighGrayLevelZoneEmphasis wavelet_HHL_glszm_LargeAreaEmphasis wavelet_HHL_glszm_LargeAreaHighGrayLevelEmphasis wavelet_HHL_glszm_LargeAreaLowGrayLevelEmphasis wavelet_HHL_glszm_LowGrayLevelZoneEmphasis wavelet_HHL_glszm_SizeZoneNonUniformity wavelet_HHL_glszm_SizeZoneNonUniformityNormalized wavelet_HHL_glszm_SmallAreaEmphasis wavelet_HHL_glszm_SmallAreaHighGrayLevelEmphasis wavelet_HHL_glszm_SmallAreaLowGrayLevelEmphasis wavelet_HHL_glszm_ZoneEntropy wavelet_HHL_glszm_ZonePercentage wavelet_HHL_glszm_ZoneVariance wavelet_HHL_ngtdm_Busyness wavelet_HHL_ngtdm_Coarseness wavelet_HHL_ngtdm_Complexity wavelet_HHL_ngtdm_Contrast wavelet_HHL_ngtdm_Strength wavelet_HLH_firstorder_10Percentile wavelet_HLH_firstorder_90Percentile wavelet_HLH_firstorder_Energy wavelet_HLH_firstorder_Entropy wavelet_HLH_firstorder_InterquartileRange wavelet_HLH_firstorder_Kurtosis wavelet_HLH_firstorder_Maximum wavelet_HLH_firstorder_Mean wavelet_HLH_firstorder_MeanAbsoluteDeviation wavelet_HLH_firstorder_Median wavelet_HLH_firstorder_Minimum wavelet_HLH_firstorder_Range wavelet_HLH_firstorder_RobustMeanAbsoluteDeviation wavelet_HLH_firstorder_RootMeanSquared wavelet_HLH_firstorder_Skewness wavelet_HLH_firstorder_TotalEnergy wavelet_HLH_firstorder_Uniformity wavelet_HLH_firstorder_Variance wavelet_HLH_glcm_Autocorrelation wavelet_HLH_glcm_ClusterProminence wavelet_HLH_glcm_ClusterShade wavelet_HLH_glcm_ClusterTendency wavelet_HLH_glcm_Contrast wavelet_HLH_glcm_Correlation wavelet_HLH_glcm_DifferenceAverage wavelet_HLH_glcm_DifferenceEntropy wavelet_HLH_glcm_DifferenceVariance wavelet_HLH_glcm_Id wavelet_HLH_glcm_Idm wavelet_HLH_glcm_Idmn wavelet_HLH_glcm_Idn wavelet_HLH_glcm_Imc1 wavelet_HLH_glcm_Imc2 wavelet_HLH_glcm_InverseVariance wavelet_HLH_glcm_JointAverage wavelet_HLH_glcm_JointEnergy wavelet_HLH_glcm_JointEntropy wavelet_HLH_glcm_MaximumProbability wavelet_HLH_glcm_SumEntropy wavelet_HLH_glcm_SumSquares wavelet_HLH_gldm_DependenceEntropy wavelet_HLH_gldm_DependenceNonUniformity wavelet_HLH_gldm_DependenceNonUniformityNormalized wavelet_HLH_gldm_DependenceVariance wavelet_HLH_gldm_GrayLevelNonUniformity wavelet_HLH_gldm_GrayLevelVariance wavelet_HLH_gldm_HighGrayLevelEmphasis wavelet_HLH_gldm_LargeDependenceEmphasis wavelet_HLH_gldm_LargeDependenceHighGrayLevelEmphasis wavelet_HLH_gldm_LargeDependenceLowGrayLevelEmphasis wavelet_HLH_gldm_LowGrayLevelEmphasis wavelet_HLH_gldm_SmallDependenceEmphasis wavelet_HLH_gldm_SmallDependenceHighGrayLevelEmphasis wavelet_HLH_gldm_SmallDependenceLowGrayLevelEmphasis wavelet_HLH_glrlm_GrayLevelNonUniformity wavelet_HLH_glrlm_GrayLevelNonUniformityNormalized wavelet_HLH_glrlm_GrayLevelVariance wavelet_HLH_glrlm_HighGrayLevelRunEmphasis wavelet_HLH_glrlm_LongRunEmphasis wavelet_HLH_glrlm_LongRunHighGrayLevelEmphasis wavelet_HLH_glrlm_LongRunLowGrayLevelEmphasis wavelet_HLH_glrlm_LowGrayLevelRunEmphasis wavelet_HLH_glrlm_RunEntropy wavelet_HLH_glrlm_RunLengthNonUniformity wavelet_HLH_glrlm_RunLengthNonUniformityNormalized wavelet_HLH_glrlm_RunPercentage wavelet_HLH_glrlm_RunVariance wavelet_HLH_glrlm_ShortRunEmphasis wavelet_HLH_glrlm_ShortRunHighGrayLevelEmphasis wavelet_HLH_glrlm_ShortRunLowGrayLevelEmphasis wavelet_HLH_glszm_GrayLevelNonUniformity wavelet_HLH_glszm_GrayLevelNonUniformityNormalized wavelet_HLH_glszm_GrayLevelVariance wavelet_HLH_glszm_HighGrayLevelZoneEmphasis wavelet_HLH_glszm_LargeAreaEmphasis wavelet_HLH_glszm_LargeAreaHighGrayLevelEmphasis wavelet_HLH_glszm_LargeAreaLowGrayLevelEmphasis wavelet_HLH_glszm_LowGrayLevelZoneEmphasis wavelet_HLH_glszm_SizeZoneNonUniformity wavelet_HLH_glszm_SizeZoneNonUniformityNormalized wavelet_HLH_glszm_SmallAreaEmphasis wavelet_HLH_glszm_SmallAreaHighGrayLevelEmphasis wavelet_HLH_glszm_SmallAreaLowGrayLevelEmphasis wavelet_HLH_glszm_ZoneEntropy wavelet_HLH_glszm_ZonePercentage wavelet_HLH_glszm_ZoneVariance wavelet_HLH_ngtdm_Busyness wavelet_HLH_ngtdm_Coarseness wavelet_HLH_ngtdm_Complexity wavelet_HLH_ngtdm_Contrast wavelet_HLH_ngtdm_Strength wavelet_HLL_firstorder_10Percentile wavelet_HLL_firstorder_90Percentile wavelet_HLL_firstorder_Energy wavelet_HLL_firstorder_Entropy wavelet_HLL_firstorder_InterquartileRange wavelet_HLL_firstorder_Kurtosis wavelet_HLL_firstorder_Maximum wavelet_HLL_firstorder_Mean wavelet_HLL_firstorder_MeanAbsoluteDeviation wavelet_HLL_firstorder_Median wavelet_HLL_firstorder_Minimum wavelet_HLL_firstorder_Range wavelet_HLL_firstorder_RobustMeanAbsoluteDeviation wavelet_HLL_firstorder_RootMeanSquared wavelet_HLL_firstorder_Skewness wavelet_HLL_firstorder_TotalEnergy wavelet_HLL_firstorder_Uniformity wavelet_HLL_firstorder_Variance wavelet_HLL_glcm_Autocorrelation wavelet_HLL_glcm_ClusterProminence wavelet_HLL_glcm_ClusterShade wavelet_HLL_glcm_ClusterTendency wavelet_HLL_glcm_Contrast wavelet_HLL_glcm_Correlation wavelet_HLL_glcm_DifferenceAverage wavelet_HLL_glcm_DifferenceEntropy wavelet_HLL_glcm_DifferenceVariance wavelet_HLL_glcm_Id wavelet_HLL_glcm_Idm wavelet_HLL_glcm_Idmn wavelet_HLL_glcm_Idn wavelet_HLL_glcm_Imc1 wavelet_HLL_glcm_Imc2 wavelet_HLL_glcm_InverseVariance wavelet_HLL_glcm_JointAverage wavelet_HLL_glcm_JointEnergy wavelet_HLL_glcm_JointEntropy wavelet_HLL_glcm_MaximumProbability wavelet_HLL_glcm_SumEntropy wavelet_HLL_glcm_SumSquares wavelet_HLL_gldm_DependenceEntropy wavelet_HLL_gldm_DependenceNonUniformity wavelet_HLL_gldm_DependenceNonUniformityNormalized wavelet_HLL_gldm_DependenceVariance wavelet_HLL_gldm_GrayLevelNonUniformity wavelet_HLL_gldm_GrayLevelVariance wavelet_HLL_gldm_HighGrayLevelEmphasis wavelet_HLL_gldm_LargeDependenceEmphasis wavelet_HLL_gldm_LargeDependenceHighGrayLevelEmphasis wavelet_HLL_gldm_LargeDependenceLowGrayLevelEmphasis wavelet_HLL_gldm_LowGrayLevelEmphasis wavelet_HLL_gldm_SmallDependenceEmphasis wavelet_HLL_gldm_SmallDependenceHighGrayLevelEmphasis wavelet_HLL_gldm_SmallDependenceLowGrayLevelEmphasis wavelet_HLL_glrlm_GrayLevelNonUniformity wavelet_HLL_glrlm_GrayLevelNonUniformityNormalized wavelet_HLL_glrlm_GrayLevelVariance wavelet_HLL_glrlm_HighGrayLevelRunEmphasis wavelet_HLL_glrlm_LongRunEmphasis wavelet_HLL_glrlm_LongRunHighGrayLevelEmphasis wavelet_HLL_glrlm_LongRunLowGrayLevelEmphasis wavelet_HLL_glrlm_LowGrayLevelRunEmphasis wavelet_HLL_glrlm_RunEntropy wavelet_HLL_glrlm_RunLengthNonUniformity wavelet_HLL_glrlm_RunLengthNonUniformityNormalized wavelet_HLL_glrlm_RunPercentage wavelet_HLL_glrlm_RunVariance wavelet_HLL_glrlm_ShortRunEmphasis wavelet_HLL_glrlm_ShortRunHighGrayLevelEmphasis wavelet_HLL_glrlm_ShortRunLowGrayLevelEmphasis wavelet_HLL_glszm_GrayLevelNonUniformity wavelet_HLL_glszm_GrayLevelNonUniformityNormalized wavelet_HLL_glszm_GrayLevelVariance wavelet_HLL_glszm_HighGrayLevelZoneEmphasis wavelet_HLL_glszm_LargeAreaEmphasis wavelet_HLL_glszm_LargeAreaHighGrayLevelEmphasis wavelet_HLL_glszm_LargeAreaLowGrayLevelEmphasis wavelet_HLL_glszm_LowGrayLevelZoneEmphasis wavelet_HLL_glszm_SizeZoneNonUniformity wavelet_HLL_glszm_SizeZoneNonUniformityNormalized wavelet_HLL_glszm_SmallAreaEmphasis wavelet_HLL_glszm_SmallAreaHighGrayLevelEmphasis wavelet_HLL_glszm_SmallAreaLowGrayLevelEmphasis wavelet_HLL_glszm_ZoneEntropy wavelet_HLL_glszm_ZonePercentage wavelet_HLL_glszm_ZoneVariance wavelet_HLL_ngtdm_Busyness wavelet_HLL_ngtdm_Coarseness wavelet_HLL_ngtdm_Complexity wavelet_HLL_ngtdm_Contrast wavelet_HLL_ngtdm_Strength wavelet_LHH_firstorder_10Percentile wavelet_LHH_firstorder_90Percentile wavelet_LHH_firstorder_Energy wavelet_LHH_firstorder_Entropy wavelet_LHH_firstorder_InterquartileRange wavelet_LHH_firstorder_Kurtosis wavelet_LHH_firstorder_Maximum wavelet_LHH_firstorder_Mean wavelet_LHH_firstorder_MeanAbsoluteDeviation wavelet_LHH_firstorder_Median wavelet_LHH_firstorder_Minimum wavelet_LHH_firstorder_Range wavelet_LHH_firstorder_RobustMeanAbsoluteDeviation wavelet_LHH_firstorder_RootMeanSquared wavelet_LHH_firstorder_Skewness wavelet_LHH_firstorder_TotalEnergy wavelet_LHH_firstorder_Uniformity wavelet_LHH_firstorder_Variance wavelet_LHH_glcm_Autocorrelation wavelet_LHH_glcm_ClusterProminence wavelet_LHH_glcm_ClusterShade wavelet_LHH_glcm_ClusterTendency wavelet_LHH_glcm_Contrast wavelet_LHH_glcm_Correlation wavelet_LHH_glcm_DifferenceAverage wavelet_LHH_glcm_DifferenceEntropy wavelet_LHH_glcm_DifferenceVariance wavelet_LHH_glcm_Id wavelet_LHH_glcm_Idm wavelet_LHH_glcm_Idmn wavelet_LHH_glcm_Idn wavelet_LHH_glcm_Imc1 wavelet_LHH_glcm_Imc2 wavelet_LHH_glcm_InverseVariance wavelet_LHH_glcm_JointAverage wavelet_LHH_glcm_JointEnergy wavelet_LHH_glcm_JointEntropy wavelet_LHH_glcm_MaximumProbability wavelet_LHH_glcm_SumEntropy wavelet_LHH_glcm_SumSquares wavelet_LHH_gldm_DependenceEntropy wavelet_LHH_gldm_DependenceNonUniformity wavelet_LHH_gldm_DependenceNonUniformityNormalized wavelet_LHH_gldm_DependenceVariance wavelet_LHH_gldm_GrayLevelNonUniformity wavelet_LHH_gldm_GrayLevelVariance wavelet_LHH_gldm_HighGrayLevelEmphasis wavelet_LHH_gldm_LargeDependenceEmphasis wavelet_LHH_gldm_LargeDependenceHighGrayLevelEmphasis wavelet_LHH_gldm_LargeDependenceLowGrayLevelEmphasis wavelet_LHH_gldm_LowGrayLevelEmphasis wavelet_LHH_gldm_SmallDependenceEmphasis wavelet_LHH_gldm_SmallDependenceHighGrayLevelEmphasis wavelet_LHH_gldm_SmallDependenceLowGrayLevelEmphasis wavelet_LHH_glrlm_GrayLevelNonUniformity wavelet_LHH_glrlm_GrayLevelNonUniformityNormalized wavelet_LHH_glrlm_GrayLevelVariance wavelet_LHH_glrlm_HighGrayLevelRunEmphasis wavelet_LHH_glrlm_LongRunEmphasis wavelet_LHH_glrlm_LongRunHighGrayLevelEmphasis wavelet_LHH_glrlm_LongRunLowGrayLevelEmphasis wavelet_LHH_glrlm_LowGrayLevelRunEmphasis wavelet_LHH_glrlm_RunEntropy wavelet_LHH_glrlm_RunLengthNonUniformity wavelet_LHH_glrlm_RunLengthNonUniformityNormalized wavelet_LHH_glrlm_RunPercentage wavelet_LHH_glrlm_RunVariance wavelet_LHH_glrlm_ShortRunEmphasis wavelet_LHH_glrlm_ShortRunHighGrayLevelEmphasis wavelet_LHH_glrlm_ShortRunLowGrayLevelEmphasis wavelet_LHH_glszm_GrayLevelNonUniformity wavelet_LHH_glszm_GrayLevelNonUniformityNormalized wavelet_LHH_glszm_GrayLevelVariance wavelet_LHH_glszm_HighGrayLevelZoneEmphasis wavelet_LHH_glszm_LargeAreaEmphasis wavelet_LHH_glszm_LargeAreaHighGrayLevelEmphasis wavelet_LHH_glszm_LargeAreaLowGrayLevelEmphasis wavelet_LHH_glszm_LowGrayLevelZoneEmphasis wavelet_LHH_glszm_SizeZoneNonUniformity wavelet_LHH_glszm_SizeZoneNonUniformityNormalized wavelet_LHH_glszm_SmallAreaEmphasis wavelet_LHH_glszm_SmallAreaHighGrayLevelEmphasis wavelet_LHH_glszm_SmallAreaLowGrayLevelEmphasis wavelet_LHH_glszm_ZoneEntropy wavelet_LHH_glszm_ZonePercentage wavelet_LHH_glszm_ZoneVariance wavelet_LHH_ngtdm_Busyness wavelet_LHH_ngtdm_Coarseness wavelet_LHH_ngtdm_Complexity wavelet_LHH_ngtdm_Contrast wavelet_LHH_ngtdm_Strength wavelet_LHL_firstorder_10Percentile wavelet_LHL_firstorder_90Percentile wavelet_LHL_firstorder_Energy wavelet_LHL_firstorder_Entropy wavelet_LHL_firstorder_InterquartileRange wavelet_LHL_firstorder_Kurtosis wavelet_LHL_firstorder_Maximum wavelet_LHL_firstorder_Mean wavelet_LHL_firstorder_MeanAbsoluteDeviation wavelet_LHL_firstorder_Median wavelet_LHL_firstorder_Minimum wavelet_LHL_firstorder_Range wavelet_LHL_firstorder_RobustMeanAbsoluteDeviation wavelet_LHL_firstorder_RootMeanSquared wavelet_LHL_firstorder_Skewness wavelet_LHL_firstorder_TotalEnergy wavelet_LHL_firstorder_Uniformity wavelet_LHL_firstorder_Variance wavelet_LHL_glcm_Autocorrelation wavelet_LHL_glcm_ClusterProminence wavelet_LHL_glcm_ClusterShade wavelet_LHL_glcm_ClusterTendency wavelet_LHL_glcm_Contrast wavelet_LHL_glcm_Correlation wavelet_LHL_glcm_DifferenceAverage wavelet_LHL_glcm_DifferenceEntropy wavelet_LHL_glcm_DifferenceVariance wavelet_LHL_glcm_Id wavelet_LHL_glcm_Idm wavelet_LHL_glcm_Idmn wavelet_LHL_glcm_Idn wavelet_LHL_glcm_Imc1 wavelet_LHL_glcm_Imc2 wavelet_LHL_glcm_InverseVariance wavelet_LHL_glcm_JointAverage wavelet_LHL_glcm_JointEnergy wavelet_LHL_glcm_JointEntropy wavelet_LHL_glcm_MaximumProbability wavelet_LHL_glcm_SumEntropy wavelet_LHL_glcm_SumSquares wavelet_LHL_gldm_DependenceEntropy wavelet_LHL_gldm_DependenceNonUniformity wavelet_LHL_gldm_DependenceNonUniformityNormalized wavelet_LHL_gldm_DependenceVariance wavelet_LHL_gldm_GrayLevelNonUniformity wavelet_LHL_gldm_GrayLevelVariance wavelet_LHL_gldm_HighGrayLevelEmphasis wavelet_LHL_gldm_LargeDependenceEmphasis wavelet_LHL_gldm_LargeDependenceHighGrayLevelEmphasis wavelet_LHL_gldm_LargeDependenceLowGrayLevelEmphasis wavelet_LHL_gldm_LowGrayLevelEmphasis wavelet_LHL_gldm_SmallDependenceEmphasis wavelet_LHL_gldm_SmallDependenceHighGrayLevelEmphasis wavelet_LHL_gldm_SmallDependenceLowGrayLevelEmphasis wavelet_LHL_glrlm_GrayLevelNonUniformity wavelet_LHL_glrlm_GrayLevelNonUniformityNormalized wavelet_LHL_glrlm_GrayLevelVariance wavelet_LHL_glrlm_HighGrayLevelRunEmphasis wavelet_LHL_glrlm_LongRunEmphasis wavelet_LHL_glrlm_LongRunHighGrayLevelEmphasis wavelet_LHL_glrlm_LongRunLowGrayLevelEmphasis wavelet_LHL_glrlm_LowGrayLevelRunEmphasis wavelet_LHL_glrlm_RunEntropy wavelet_LHL_glrlm_RunLengthNonUniformity wavelet_LHL_glrlm_RunLengthNonUniformityNormalized wavelet_LHL_glrlm_RunPercentage wavelet_LHL_glrlm_RunVariance wavelet_LHL_glrlm_ShortRunEmphasis wavelet_LHL_glrlm_ShortRunHighGrayLevelEmphasis wavelet_LHL_glrlm_ShortRunLowGrayLevelEmphasis wavelet_LHL_glszm_GrayLevelNonUniformity wavelet_LHL_glszm_GrayLevelNonUniformityNormalized wavelet_LHL_glszm_GrayLevelVariance wavelet_LHL_glszm_HighGrayLevelZoneEmphasis wavelet_LHL_glszm_LargeAreaEmphasis wavelet_LHL_glszm_LargeAreaHighGrayLevelEmphasis wavelet_LHL_glszm_LargeAreaLowGrayLevelEmphasis wavelet_LHL_glszm_LowGrayLevelZoneEmphasis wavelet_LHL_glszm_SizeZoneNonUniformity wavelet_LHL_glszm_SizeZoneNonUniformityNormalized wavelet_LHL_glszm_SmallAreaEmphasis wavelet_LHL_glszm_SmallAreaHighGrayLevelEmphasis wavelet_LHL_glszm_SmallAreaLowGrayLevelEmphasis wavelet_LHL_glszm_ZoneEntropy wavelet_LHL_glszm_ZonePercentage wavelet_LHL_glszm_ZoneVariance wavelet_LHL_ngtdm_Busyness wavelet_LHL_ngtdm_Coarseness wavelet_LHL_ngtdm_Complexity wavelet_LHL_ngtdm_Contrast wavelet_LHL_ngtdm_Strength wavelet_LLH_firstorder_10Percentile wavelet_LLH_firstorder_90Percentile wavelet_LLH_firstorder_Energy wavelet_LLH_firstorder_Entropy wavelet_LLH_firstorder_InterquartileRange wavelet_LLH_firstorder_Kurtosis wavelet_LLH_firstorder_Maximum wavelet_LLH_firstorder_Mean wavelet_LLH_firstorder_MeanAbsoluteDeviation wavelet_LLH_firstorder_Median wavelet_LLH_firstorder_Minimum wavelet_LLH_firstorder_Range wavelet_LLH_firstorder_RobustMeanAbsoluteDeviation wavelet_LLH_firstorder_RootMeanSquared wavelet_LLH_firstorder_Skewness wavelet_LLH_firstorder_TotalEnergy wavelet_LLH_firstorder_Uniformity wavelet_LLH_firstorder_Variance wavelet_LLH_glcm_Autocorrelation wavelet_LLH_glcm_ClusterProminence wavelet_LLH_glcm_ClusterShade wavelet_LLH_glcm_ClusterTendency wavelet_LLH_glcm_Contrast wavelet_LLH_glcm_Correlation wavelet_LLH_glcm_DifferenceAverage wavelet_LLH_glcm_DifferenceEntropy wavelet_LLH_glcm_DifferenceVariance wavelet_LLH_glcm_Id wavelet_LLH_glcm_Idm wavelet_LLH_glcm_Idmn wavelet_LLH_glcm_Idn wavelet_LLH_glcm_Imc1 wavelet_LLH_glcm_Imc2 wavelet_LLH_glcm_InverseVariance wavelet_LLH_glcm_JointAverage wavelet_LLH_glcm_JointEnergy wavelet_LLH_glcm_JointEntropy wavelet_LLH_glcm_MaximumProbability wavelet_LLH_glcm_SumEntropy wavelet_LLH_glcm_SumSquares wavelet_LLH_gldm_DependenceEntropy wavelet_LLH_gldm_DependenceNonUniformity wavelet_LLH_gldm_DependenceNonUniformityNormalized wavelet_LLH_gldm_DependenceVariance wavelet_LLH_gldm_GrayLevelNonUniformity wavelet_LLH_gldm_GrayLevelVariance wavelet_LLH_gldm_HighGrayLevelEmphasis wavelet_LLH_gldm_LargeDependenceEmphasis wavelet_LLH_gldm_LargeDependenceHighGrayLevelEmphasis wavelet_LLH_gldm_LargeDependenceLowGrayLevelEmphasis wavelet_LLH_gldm_LowGrayLevelEmphasis wavelet_LLH_gldm_SmallDependenceEmphasis wavelet_LLH_gldm_SmallDependenceHighGrayLevelEmphasis wavelet_LLH_gldm_SmallDependenceLowGrayLevelEmphasis wavelet_LLH_glrlm_GrayLevelNonUniformity wavelet_LLH_glrlm_GrayLevelNonUniformityNormalized wavelet_LLH_glrlm_GrayLevelVariance wavelet_LLH_glrlm_HighGrayLevelRunEmphasis wavelet_LLH_glrlm_LongRunEmphasis wavelet_LLH_glrlm_LongRunHighGrayLevelEmphasis wavelet_LLH_glrlm_LongRunLowGrayLevelEmphasis wavelet_LLH_glrlm_LowGrayLevelRunEmphasis wavelet_LLH_glrlm_RunEntropy wavelet_LLH_glrlm_RunLengthNonUniformity wavelet_LLH_glrlm_RunLengthNonUniformityNormalized wavelet_LLH_glrlm_RunPercentage wavelet_LLH_glrlm_RunVariance wavelet_LLH_glrlm_ShortRunEmphasis wavelet_LLH_glrlm_ShortRunHighGrayLevelEmphasis wavelet_LLH_glrlm_ShortRunLowGrayLevelEmphasis wavelet_LLH_glszm_GrayLevelNonUniformity wavelet_LLH_glszm_GrayLevelNonUniformityNormalized wavelet_LLH_glszm_GrayLevelVariance wavelet_LLH_glszm_HighGrayLevelZoneEmphasis wavelet_LLH_glszm_LargeAreaEmphasis wavelet_LLH_glszm_LargeAreaHighGrayLevelEmphasis wavelet_LLH_glszm_LargeAreaLowGrayLevelEmphasis wavelet_LLH_glszm_LowGrayLevelZoneEmphasis wavelet_LLH_glszm_SizeZoneNonUniformity wavelet_LLH_glszm_SizeZoneNonUniformityNormalized wavelet_LLH_glszm_SmallAreaEmphasis wavelet_LLH_glszm_SmallAreaHighGrayLevelEmphasis wavelet_LLH_glszm_SmallAreaLowGrayLevelEmphasis wavelet_LLH_glszm_ZoneEntropy wavelet_LLH_glszm_ZonePercentage wavelet_LLH_glszm_ZoneVariance wavelet_LLH_ngtdm_Busyness wavelet_LLH_ngtdm_Coarseness wavelet_LLH_ngtdm_Complexity wavelet_LLH_ngtdm_Contrast wavelet_LLH_ngtdm_Strength wavelet_LLL_firstorder_10Percentile wavelet_LLL_firstorder_90Percentile wavelet_LLL_firstorder_Energy wavelet_LLL_firstorder_Entropy wavelet_LLL_firstorder_InterquartileRange wavelet_LLL_firstorder_Kurtosis wavelet_LLL_firstorder_Maximum wavelet_LLL_firstorder_Mean wavelet_LLL_firstorder_MeanAbsoluteDeviation wavelet_LLL_firstorder_Median wavelet_LLL_firstorder_Minimum wavelet_LLL_firstorder_Range wavelet_LLL_firstorder_RobustMeanAbsoluteDeviation wavelet_LLL_firstorder_RootMeanSquared wavelet_LLL_firstorder_Skewness wavelet_LLL_firstorder_TotalEnergy wavelet_LLL_firstorder_Uniformity wavelet_LLL_firstorder_Variance wavelet_LLL_glcm_Autocorrelation wavelet_LLL_glcm_ClusterProminence wavelet_LLL_glcm_ClusterShade wavelet_LLL_glcm_ClusterTendency wavelet_LLL_glcm_Contrast wavelet_LLL_glcm_Correlation wavelet_LLL_glcm_DifferenceAverage wavelet_LLL_glcm_DifferenceEntropy wavelet_LLL_glcm_DifferenceVariance wavelet_LLL_glcm_Id wavelet_LLL_glcm_Idm wavelet_LLL_glcm_Idmn wavelet_LLL_glcm_Idn wavelet_LLL_glcm_Imc1 wavelet_LLL_glcm_Imc2 wavelet_LLL_glcm_InverseVariance wavelet_LLL_glcm_JointAverage wavelet_LLL_glcm_JointEnergy wavelet_LLL_glcm_JointEntropy wavelet_LLL_glcm_MaximumProbability wavelet_LLL_glcm_SumEntropy wavelet_LLL_glcm_SumSquares wavelet_LLL_gldm_DependenceEntropy wavelet_LLL_gldm_DependenceNonUniformity wavelet_LLL_gldm_DependenceNonUniformityNormalized wavelet_LLL_gldm_DependenceVariance wavelet_LLL_gldm_GrayLevelNonUniformity wavelet_LLL_gldm_GrayLevelVariance wavelet_LLL_gldm_HighGrayLevelEmphasis wavelet_LLL_gldm_LargeDependenceEmphasis wavelet_LLL_gldm_LargeDependenceHighGrayLevelEmphasis wavelet_LLL_gldm_LargeDependenceLowGrayLevelEmphasis wavelet_LLL_gldm_LowGrayLevelEmphasis wavelet_LLL_gldm_SmallDependenceEmphasis wavelet_LLL_gldm_SmallDependenceHighGrayLevelEmphasis wavelet_LLL_gldm_SmallDependenceLowGrayLevelEmphasis wavelet_LLL_glrlm_GrayLevelNonUniformity wavelet_LLL_glrlm_GrayLevelNonUniformityNormalized wavelet_LLL_glrlm_GrayLevelVariance wavelet_LLL_glrlm_HighGrayLevelRunEmphasis wavelet_LLL_glrlm_LongRunEmphasis wavelet_LLL_glrlm_LongRunHighGrayLevelEmphasis wavelet_LLL_glrlm_LongRunLowGrayLevelEmphasis wavelet_LLL_glrlm_LowGrayLevelRunEmphasis wavelet_LLL_glrlm_RunEntropy wavelet_LLL_glrlm_RunLengthNonUniformity wavelet_LLL_glrlm_RunLengthNonUniformityNormalized wavelet_LLL_glrlm_RunPercentage wavelet_LLL_glrlm_RunVariance wavelet_LLL_glrlm_ShortRunEmphasis wavelet_LLL_glrlm_ShortRunHighGrayLevelEmphasis wavelet_LLL_glrlm_ShortRunLowGrayLevelEmphasis wavelet_LLL_glszm_GrayLevelNonUniformity wavelet_LLL_glszm_GrayLevelNonUniformityNormalized wavelet_LLL_glszm_GrayLevelVariance wavelet_LLL_glszm_HighGrayLevelZoneEmphasis wavelet_LLL_glszm_LargeAreaEmphasis wavelet_LLL_glszm_LargeAreaHighGrayLevelEmphasis wavelet_LLL_glszm_LargeAreaLowGrayLevelEmphasis wavelet_LLL_glszm_LowGrayLevelZoneEmphasis wavelet_LLL_glszm_SizeZoneNonUniformity wavelet_LLL_glszm_SizeZoneNonUniformityNormalized wavelet_LLL_glszm_SmallAreaEmphasis wavelet_LLL_glszm_SmallAreaHighGrayLevelEmphasis wavelet_LLL_glszm_SmallAreaLowGrayLevelEmphasis wavelet_LLL_glszm_ZoneEntropy wavelet_LLL_glszm_ZonePercentage wavelet_LLL_glszm_ZoneVariance wavelet_LLL_ngtdm_Busyness wavelet_LLL_ngtdm_Coarseness wavelet_LLL_ngtdm_Complexity wavelet_LLL_ngtdm_Contrast wavelet_LLL_ngtdm_Strength
